# Optimization of Genomic Breeding Value Estimation Model for Abdominal Fat Traits Based on Machine Learning

**DOI:** 10.3390/ani15192843

**Published:** 2025-09-29

**Authors:** Hengcong Chen, Dachang Dou, Min Lu, Xintong Liu, Cheng Chang, Fuyang Zhang, Shengwei Yang, Zhiping Cao, Peng Luan, Yumao Li, Hui Zhang

**Affiliations:** Key Laboratory of Chicken Genetics and Breeding, Ministry of Agriculture and Rural Affairs, College of Animal Science and Technology, Northeast Agricultural University, Harbin 150030, China; 15610303963@163.com (H.C.); doudachang@163.com (D.D.); 18254839393@163.com (M.L.); lxt202720396@163.com (X.L.); b230501003@neau.edu.cn (C.C.); s220501010@neau.edu.cn (F.Z.); yangshengwei@neau.edu.cn (S.Y.); caozhiping@neau.edu.cn (Z.C.); luan0901@neau.edu.cn (P.L.); liyumao@neau.edu.cn (Y.L.)

**Keywords:** genomic selection/prediction, breeding value estimation, abdominal fat, machine learning, DAWSELF

## Abstract

Abdominal fat is a key indicator of chicken meat quality. Excessive deposition not only reduces meat quality but also decreases feed conversion efficiency, making the breeding of low-abdominal-fat strains economically important. Genomic selection (GS) uses information from genome-wide association studies (GWASs) and high-throughput sequencing data. It estimates genomic breeding values (GEBVs) from genotypes, which enables early and precise selection. Given that abdominal fat is a polygenic trait controlled by numerous small-effect loci, this study combined population genetic analyses with machine learning (ML)-based feature selection. Relevant single-nucleotide polymorphisms (SNPs) were first identified using a combined GWAS and linkage disequilibrium (LD) approach, followed by a two-stage feature selection process—Lasso for dimensionality reduction and recursive feature elimination (RFE) for refinement—to generate the model input set. We evaluated multiple machine learning models for predicting genomic estimated breeding values (GEBVs). The results showed that linear models and certain nonlinear models achieved higher accuracy and were well suited as base learners for ensemble methods. Building on these findings, we developed a Dynamic Adaptive Weighted Stacking Ensemble Learning Framework (DAWSELF), which applies dynamic weighting and voting to heterogeneous base learners and integrates them layer by layer, with Ridge serving as the meta-learner. In three independent validation populations, DAWSELF consistently outperformed individual models and conventional stacking frameworks in prediction accuracy. This work establishes an efficient GEBV prediction framework for complex traits such as chicken abdominal fat and provides a reusable SNP feature selection strategy, offering practical value for enhancing the precision of poultry breeding and improving product quality.

## Simple Summary

Excessive abdominal fat in chickens not only lowers meat quality but also wastes feed, which reduces overall breeding efficiency. Because abdominal fat is influenced by many genes, it is difficult to predict accurately with traditional methods. In this study, we used modern data analysis and artificial intelligence techniques to improve prediction. We first searched the chicken genome to find genetic markers related to abdominal fat and then gradually narrowed down the most useful ones. Next, we tested different computer models and developed a new method that combines the strengths of several models. This method consistently gave more accurate predictions of fat content across different chicken populations. Our work provides poultry breeders with practical tools to select chickens more precisely, helping improve meat quality and breeding efficiency.

## 1. Introduction

Due to its low fat content, high protein value, and easy digestibility, chicken has become a major source of animal protein worldwide, holding an irreplaceable role in the human diet [1]. As living standards continue to rise, consumer demand for chicken has shifted beyond quantity to emphasize quality and health attributes. Abdominal fat is the main fat storage site in chickens, and its deposition strongly affects meat quality, feed efficiency, and economic returns [2,3]. Consequently, precisely regulating abdominal fat deposition to improve meat quality and reduce production costs has become an urgent priority in modern poultry breeding.

In traditional poultry breeding, trait improvement has largely relied on phenotypic records and pedigree information [2]. For traits such as abdominal fat weight, which require slaughter-based measurements or incur high measurement costs, it is often challenging to obtain sufficient phenotypic data in a timely manner, thereby slowing the breeding process. The advent of genomic selection (GS) has provided a novel solution to this problem. First proposed by Meuwissen et al. [3] in 2001, GS leverages a large number of single-nucleotide polymorphism (SNP) markers across the genome to establish statistical models linking genotypes to phenotypes, enabling the prediction of genomic estimated breeding values (GEBVs) for individuals. Unlike marker-assisted selection, GS enables early and accurate prediction, shortens generation intervals, and is particularly useful for traits with low heritability or high measurement costs [4].

The development of genomic selection (GS) models has progressed through several stages. Initially, linear models such as genomic best linear unbiased prediction (GBLUP) [5] and ridge regression BLUP (RR-BLUP) [6] became the predominant approaches. These methods assume that all SNPs contribute equally to trait variation, offering high computational efficiency and stable performance; however, they have limited ability to fully exploit the genetic information from loci with large effects. Subsequently, Bayesian methods, including BayesA [3], BayesB [3], and BayesCπ [7], were introduced into GS. By allowing SNP effects to follow different prior distributions, these models are more effective at capturing large-effect loci, but their computational demands are substantial, particularly when the number of markers is large, resulting in prolonged processing times. More recently, advances in computational power and algorithm development have facilitated the application of machine learning methods to genomic prediction, including random forest (RF) [8], support vector machine (SVM) [9], and neural networks [10]. These approaches are capable of modeling nonlinear relationships and higher-order interactions between genotypes and traits, rely less on strict model assumptions, and demonstrate strong adaptability. Nevertheless, their performance depends heavily on appropriate parameter tuning, and they tend to be more sensitive to sample size and data quality in practical applications.

In recent years, the application of ML algorithms to genomic selection has attracted considerable attention from researchers and breeders, with various models being explored for estimating breeding values from real-world data. For example, Xiang et al. [11] used ML algorithms to identify key genomic loci related to pig production traits and incorporated them into a statistical model, reducing computational complexity while maintaining prediction accuracy. Mota et al. [12] compared ML and parametric methods for predicting feed efficiency-related traits in Nellore cattle and found that ML models generally outperformed traditional approaches, with deep learning achieving the highest accuracy. Faridi et al. [13] evaluated support vector regression (SVR) and neural networks (NNs) for predicting body weight and muscle traits in broilers, showing that both methods performed better than linear regression. Liang et al. [14] further proposed a Stacking Ensemble Learning Framework (SELF), which provided substantially higher accuracy than individual models when predicting complex traits such as egg weight and body weight.

Despite these promising advances, several challenges remain in extending ML approaches to genomic prediction. First, the genetic architecture of traits varies considerably, leading to large differences in model performance across traits. Second, some high-accuracy models lack computational efficiency, which limits their applicability in large-scale commercial breeding programs requiring rapid iteration. Lastly, the ability to efficiently extract informative markers and remove redundancy from the thousands of SNPs available remains a critical factor influencing both the accuracy and efficiency of genomic prediction.

Building on the above challenges, this study targeted chicken abdominal fat weight and utilized genome-wide SNP data to systematically compare a range of ML models with traditional GS approaches. The models were evaluated in terms of prediction accuracy, computational efficiency, and feature selection capability. Furthermore, by leveraging the strengths of different methods, we explored model optimization and feature selection strategies tailored to the genetic architecture of chicken abdominal fat traits, with the aim of providing a data-driven foundation for precision poultry breeding. The originality of this study lies in the development of DAWSELF, which not only establishes an efficient GEBV prediction framework for complex traits such as chicken abdominal fat but also introduces a reusable SNP feature selection strategy, offering practical value for enhancing the precision of poultry breeding and improving product quality.

## 2. Materials and Methods

### 2.1. Materials

In this study, a 19th-generation (G19) population of high- and low-fat bi-directional selection lines of broilers from Northeast Agricultural University (NEAU) was used as the reference population [15]. Phenotypic data, including abdominal fat weight (AFW) and pre-slaughter live weight (Weight), were recorded for all individuals at 7 weeks of age at slaughter, and genomic Deoxyribonucleic acid (DNA) was extracted for whole-genome resequencing. Three independent validation populations—the 23rd generation (G23), the 27th generation (G27), and the AA commercial line—were also included. For each validation group, phenotypic and resequencing data were collected from individuals slaughtered at 7 weeks of age. From hatching to the end of rearing, all broilers were raised under identical environmental conditions with ad libitum access to feed and water.

#### 2.1.1. Phenotypic Data

In this study, abdominal fat weight (AFW) in broilers was selected as the primary target trait. Following slaughter, the combined weight of all abdominal fat deposits and perimysogastric fat for each bird was recorded in grams (g). To account for the fixed effects of factors such as strain and sex, as well as covariates including pre-slaughter live weight, the raw phenotypic values were adjusted using the following correction model:

(1) y=μ+Line+Sex+Weight+e

In the model, y is the AFW phenotype, *μ* denotes the overall mean, Line indicates the fixed effect of strain within the high- and low-fat bi-directional selection lines, Sex represents the fixed effect of gender, Weight is included as a covariate corresponding to pre-slaughter live weight, and *e* denotes the random residual error. The adjusted phenotypes were used for genomic prediction, while the raw values were used for preliminary GWAS. Given the structural differences among populations, the number of individuals, the actual covariates applied, and the estimated heritability (*h*^2^) of AFW within each population are summarized in Table 1. Data correction and heritability estimation were performed using the ASReml (v4.2) software package [16] and the scikit-learn library in Python (3.11.0) [17].

#### 2.1.2. Genotypic Data

Two days before slaughter, venous blood samples were collected from the wings of all selected chickens. After Ethylenediaminetetraacetic acid (EDTA) anticoagulation treatment, genomic DNA was extracted from whole blood, and whole-genome resequencing was performed to obtain raw SNP data for each individual across all populations. Quality control (QC) and missing genotype imputation were conducted using VCFtools [18], PLINK [19], and Beagle [20] software following specific criteria: individuals lacking corresponding phenotypic data were excluded; SNP loci were filtered using PLINK with the parameters -geno 0.05 -maf 0.05, and only biallelic markers were retained. Missing genotypes were imputed using Beagle based on known genotype data and allele frequencies to produce a more complete dataset. After QC, the final number of SNP markers and reference genome versions used for each population were as follows: G19 contained 9,802,878 SNPs aligned to the Gallus gallus GRCg7b reference genome; G23 had 8,653,665 SNPs; G27 included 6,562,310 SNPs; and the AA population comprised 9,502,363 SNPs, both aligned to the Gallus gallus GRCg6a reference genome.

### 2.2. Methods

#### 2.2.1. Genome-Wide Association Analysis and ML Prediction Models

In this study, genome-wide association analysis study (GWAS) combined with linkage disequilibrium (LD) analysis was employed to screen the SNP markers associated with the target traits. To address the common “*n* ≪ *p*” problem of genomic prediction, markers with GWAS significance levels below the Bonferroni-corrected threshold (α = 0.05/*m*) were first selected, followed by redundancy removal through LD pruning (*r*^2^ > 0.2).

The prediction models applied in this study encompassed linear models, nonlinear models, and ensemble learning methods. Linear models included ridge regression (Ridge) [21], Least Absolute Shrinkage and Selection Operator (LASSO) [22], support vector regression (SVR) [23], and Elastic Net [24], all of which are well-suited for addressing multicollinearity in high-dimensional datasets. Nonlinear models consisted primarily of K-nearest neighbors (KNNs) [25] and artificial neural networks (ANN) [26], which are capable of capturing complex non-additive genetic effects.

Ensemble learning, an advanced approach increasingly employed to enhance predictive accuracy and generalization ability, integrates multiple base learners. In the Bagging framework, multiple base models are trained independently on bootstrap-resampled subsets of the original dataset, and their predictions are subsequently aggregated. Random Forest (RF) [27] is a representative algorithm of this category. In contrast, Boosting sequentially constructs a strong learner by iteratively combining weak learners, with each subsequent model trained on data reweighted to emphasize previously misclassified samples [28]. Gradient Boosted Decision Trees (GBDT) [29], a prominent Boosting algorithm, effectively models nonlinear relationships, performs automatic feature selection, and exhibits strong tolerance to redundant features and interactions, thereby enabling the extraction of high-quality feature subsets. On this foundation, Extreme Gradient Boosting (XGBoost, XGB) [30] and Light Gradient Boosting Machine (LightGBM, LGB) [31] have emerged as widely adopted variants. XGBoost enhances stability and generalization through mechanisms such as regularization, pruning, and missing-value handling, whereas LightGBM employs histogram-based algorithms and multi-threaded parallelization to markedly improve computational efficiency, making it particularly suitable for large-scale datasets. For both algorithms, optimal predictive performance depends on appropriate parameter tuning to balance model complexity and generalization ability.

#### 2.2.2. Feature Selection

Feature selection is a critical preprocessing step in ML, aiming to identify a subset of informative features from high-dimensional datasets that exhibit strong predictive power for the target variables. This process improves model performance, reduces computational complexity, and enhances interpretability [32]. Common approaches include the embedded method [33], wrapper method [34], and autoencoder [35]. Embedded methods, which perform feature selection during model training using measures such as feature importance, were also applied. Representative techniques include Lasso regression (a regularization-based method) and tree-based models such as Random Forest and Gradient Boosting Trees. These methods account for feature–model interactions and can yield more accurate selections, albeit at the cost of higher computational demands and model dependency. Wrapper methods, which evaluate feature subsets based on model performance, were implemented through heuristic search strategies such as recursive feature elimination (RFE), iteratively discarding features with lower contributions until an optimal subset was obtained. While highly effective, these methods are computationally expensive and impractical for ultra-high-dimensional datasets. Additionally, an unsupervised autoencoder (AE) was employed for dimensionality reduction and feature extraction. The encoder mapped high-dimensional data into a low-dimensional latent space, from which representative features were identified in conjunction with clustering analysis, facilitating the discovery of the intrinsic structure of the data.

#### 2.2.3. Dynamic Adaptive Weighted Stacking Ensemble Learning Framework

This study adopted an iterative model selection and ensemble strategy to identify the optimal architecture for predicting target traits. Given the substantial variation in breeding value estimation accuracy across models, traits, and populations, the choice of base and meta models must be tailored to specific conditions. Initially, we independently evaluated multiple candidate models—such as SVR and RF—on genomic data to identify those with superior performance in predicting abdominal fat weight.

In the first layer of DAWSELF, we directly employed both linear and nonlinear models as base learners, consistent with conventional stacking frameworks, rather than applying a custom TOP K selection function to retain only the best-performing models. This design choice was motivated by three considerations: (1) Because the first layer learns directly from raw data, its model configuration critically influences the effectiveness of the entire framework. Incorporating pre-evaluated, high-performing models ensures stability and reduces the risk of suboptimal learning. (2) Prior single-model evaluations revealed several linear and nonlinear models with consistently strong predictive ability, each capturing distinct data patterns. Their direct inclusion in the first layer guarantees sufficient representation capacity. (3) Combining linear and nonlinear models at this stage enhances model diversity, which mitigates overfitting and improves generalization to varying data distributions.

For subsequent layers, we applied a custom TOP K function to dynamically select suitable base models from a broader candidate pool. Specifically, the weighted outputs of all base models from the preceding layer were combined as new input features. Each candidate model was then evaluated on these features using five-fold cross-validation. The custom function evaluate_models generated prediction values and mean squared error (MSE) scores for each model, and the choose_topk_models function retained the K models with the lowest MSE scores, ranked in ascending order.

This dynamic TOP K strategy ensures that, at every layer, the most appropriate models are automatically selected to best fit the transformed input data, thereby improving prediction accuracy. Because the candidate pool comprises diverse model types (linear, nonlinear, and ensemble), automatic selection also preserves model diversity and strengthens generalization. Together, the use of directly selected high-performing models in the first layer and dynamic TOP K selection in subsequent layers enables DAWSELF to balance rationality and diversity throughout the modeling process. This progressive approach allows the framework to continuously refine its base model set, ultimately achieving robust and accurate prediction performance.

In each layer of DAWSELF, a dynamically weighted ensemble strategy is implemented. Each base model is assigned an adaptive weight that is continuously adjusted according to the validation set performance, thereby allowing the ensemble to better accommodate varying data distributions. To achieve this, cross-validation is employed to partition the dataset into training and validation subsets. The weight of the j-th base model in the i-th layer, denoted as *w*(*i*,*j*) is calculated as follows:

w (i, j) = 1/MSE (i, j)(2)

Here, MSE (i, j) represents the mean squared error of the j-th base model in the i-th layer, evaluated on the validation set. Consequently, models with smaller prediction errors are assigned larger weights. This weighting strategy not only reflects the individual learning capacity of each base model but also accounts for their collective contribution to the ensemble, thereby producing a more rational and balanced weight distribution.

When making predictions for new test samples, the output of layer i is constructed by horizontally concatenating the weighted outputs of all base models:

(3) $$y(i)=\sum_{j}\;w(i,j)\cdot f(i,j)(x)$$

Here, f(i,j)(x) denotes the prediction of the j-th base model in the i-th layer for input sample *x*.

DAWSELF adopts a multi-layer stacking strategy for inter-layer connections in contrast to the conventional two-layer stacking architecture. Specifically, the framework is divided into *i* layers, each comprising multiple heterogeneous base models. In the first layer, the base models learn directly from the raw input data. In subsequent layers, the base models take as input the outputs of all base models from the preceding layer, thereby establishing hierarchical connections across layers:

(4) $$x\left(i\right)=concat(f\left(i-1,1\right),f\left(i-1,2\right),\ldots ,f\left(i-1,k\right))$$

Here, *x*(*i*) denotes the input to the *i*-th layer, while *k* represents the number of base models in the (*i* − 1)-th layer, and concat refers to the horizontal concatenation of the output vectors generated by multiple base models. This layer-by-layer concatenation strategy enhances the expressive capacity of the framework and expands its parameter space. At the same time, it facilitates information sharing between higher and lower layers, thereby reducing the risk of overfitting. The final output ŷ is obtained as the weighted sum of the outputs from all base models in the last layer:

(5) $$\hat{y}=\sum_{j}\;w\left(i,j\right)\cdot f\left(i,j\right)(x\left(i\right))$$

Through its multi-level integration strategy, DAWSELF effectively harnesses the complementary strengths of diverse base models, thereby improving both the predictive accuracy and the generalization capacity of genomic breeding value estimation.

The construction of the dynamic weighted stacking ensemble learning framework primarily involves the following steps: building a list of candidate models, initializing models, selecting base models and meta-models, calculating base model weights on the training set, weighting prediction results on the test set, re-selecting the top K base models from the candidate pool for each layer, initializing, training, and predicting with the meta-model, and finally evaluating the predictive performance of the meta-model. The specific process is as follows:

(1) Define and initialize the base model list ‘models’, which includes 10 machine learning models used previously (Linear, SVR-lin, ANN, etc.). Initialize these models with the same parameters as conventional stacking models.

(2) First-layer base model selection: Directly select the best-performing linear and nonlinear models from the ‘models’ list as the first-layer base models. This provides robust initialization and model diversity for the entire framework.

(3) Perform predictions on the training set for each base model in the first layer using 5-fold cross-validation, and store the predictions in y_preds.

(4) Calculate the weights for the first-layer base models using the inverse of each model’s mean squared error (MSE) as the weight, storing them in the weights dictionary. A smaller MSE indicates better model performance, resulting in a higher weight for that model after applying 1/MSE.

(5) Make predictions for each base model in the first layer on the test set and store the predictions in the model_test_preds dictionary.

(6) Weight the predictions stored in model_test_preds using the base model weights calculated in step 4 to obtain weighted_test_preds.

(7) Subsequent layer base model selection: Stack the weighted outputs weighted_test_preds from all base models in the first layer horizontally as the new input feature X. On this new feature X, evaluate candidate models using the custom evaluate_models function. Select the top K models in descending order of performance using the choose_topk_models as the base models for the current layer.

(8) Base model weight calculation for subsequent layers: Repeat step 4, using 5-fold cross-validation to compute weights for each base model in the current layer.

(9) Test set prediction for subsequent layers: Repeat step 5 to perform test set predictions using the base models of the current layer, then perform weighted fusion.

(10) Define and initialize the meta-model meta_estimator. Use the training set prediction results of the base models from the final layer as the meta-model’s training data X_train_meta. Use the weighted test set prediction results weighted_test_preds from all layers as the meta-model’s test data.

(11) Early stopping: All iterative models employed an early stopping mechanism. Training was monitored using validation loss, and if no improvement was observed for 10 consecutive iterations, training was immediately terminated, and the model parameters were rolled back to the epoch with the lowest validation error.

(12) Finally, evaluate the meta-model’s performance, including predicted outputs, coefficient of determination (R^2^), MSE, and breeding value estimation accuracy (r).

Key points of DAWSELF are as follows: (1) base models are stacked layer by layer, with each layer automatically selecting the optimal base model; (2) base model weights are dynamically computed and weighted for prediction at each layer; (3) the meta-model models the weighted predictions from base models; and (4) the entire framework embodies the concepts of model diversity and automated selection of optimal model combinations. Through these key optimizations, DAWSELF fully leverages the advantages of ensemble learning to achieve optimal performance in genomic breeding value prediction.

### 2.3. Cross-Validation and Evaluation Metrics

In this study, the predictive performance for estimating breeding values of the target groups was evaluated using Pearson’s correlation coefficient (r) between the predicted values (y_pred_) and the observed phenotypic values (y). The coefficient r ranges from −1 to 1, where values close to 1 indicate a strong positive correlation, values near 0 suggest little or no correlation, and values approaching −1denote a strong negative correlation. As Pearson’s correlation coefficient directly reflects the degree of linear association between two variables, it is widely recognized as a reliable metric for assessing the accuracy of genomic prediction.

(6) $$r=\frac{cov\left(y,y_{pred}\right)}{\sqrt{var\left(y\right)var\left(y_{pred}\right)}}$$

In order to obtain the robustness of the evaluation results, a nested cross-validation strategy was applied to all datasets in this study. Specifically, each complete dataset was first split into a training set and a test set at a 4:1 ratio. During the training phase, hyperparameter tuning was conducted using 10-fold cross-validation within the outer training set, where the data were further divided at a 9:1 ratio. Grid search was performed on the validation folds to determine the optimal hyperparameter combination, and the model was then retrained on the entire outer training set using these selected parameters. In the testing phase, the final model’s performance was again assessed via 5-fold cross-validation on the outer test set, and the average predictive accuracy was reported. This approach mitigates the variability caused by data partitioning and enhances the reliability and robustness of the evaluation.

## 3. Results

### 3.1. Initial SNP Screening Based on GWAS and LD Methods

#### 3.1.1. Genome-Wide Association Analysis

In this study, GWAS was performed on the original genotype data of the reference population using GEMMA software [36] to identify SNPs associated with chicken abdominal fat weight. Multiple GWAS significance thresholds were evaluated (0.2, 0.1, 0.05, 0.01, 1 × 10^−3^, 1 × 10^−4^, 1 × 10^−5^), and the predictive performance of 10 candidate ML models (Linear, SVR-linear, Ridge, Elastic Net, RF, SVR-polynomial, LightGBM, XGBoost, KNN, and ANN) was used to assess the effect of SNP selection under each threshold. The GWAS *p*-value threshold yielding the highest average GEBV prediction accuracy across all models was selected as the optimal criterion for initial SNP screening related to the abdominal fat trait.

**Figure 1:**
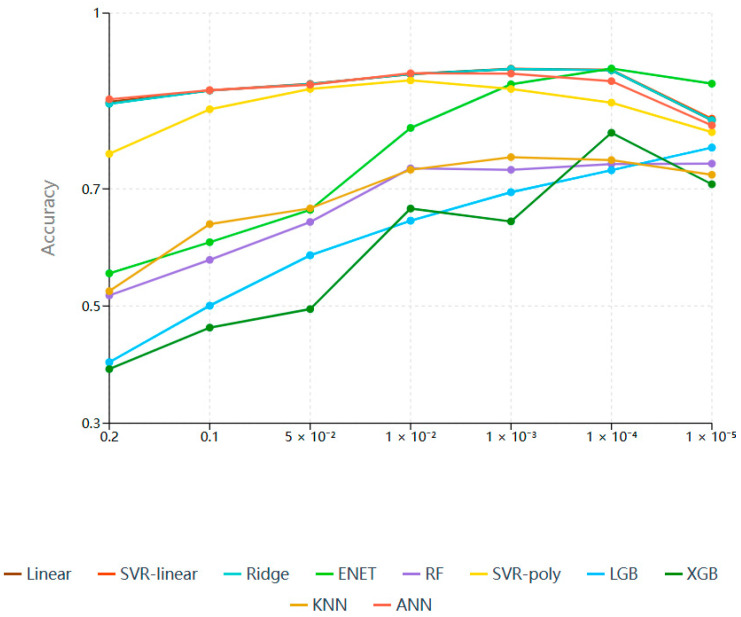
Predictive accuracy of gradient GWAS threshold screening of SNP subset in candidate ML models.

As illustrated in Figure 1 and Figure S1, for linear models (Linear, SVR-linear, Ridge, Elastic Net), prediction accuracy steadily increased as the GWAS *p*-value threshold decreased from 0.2 to 1 × 10^−4^, reaching a maximum at 1 × 10^−4^; further tightening the threshold led to a decline in accuracy. For nonlinear models (Random Forest, SVR-polynomial, LightGBM, XGBoost, KNN, and ANN), prediction accuracy generally improved with stricter thresholds, although the exact peak varied among models, with some reaching maximum accuracy at 1× 10^−4^ and others at more or less stringent cutoffs. Overall, the trend across all candidate models indicated improved prediction accuracy, with decreasing GWAS *p*-value thresholds up to a point.

#### 3.1.2. Linkage Disequilibrium

**Figure 2:**
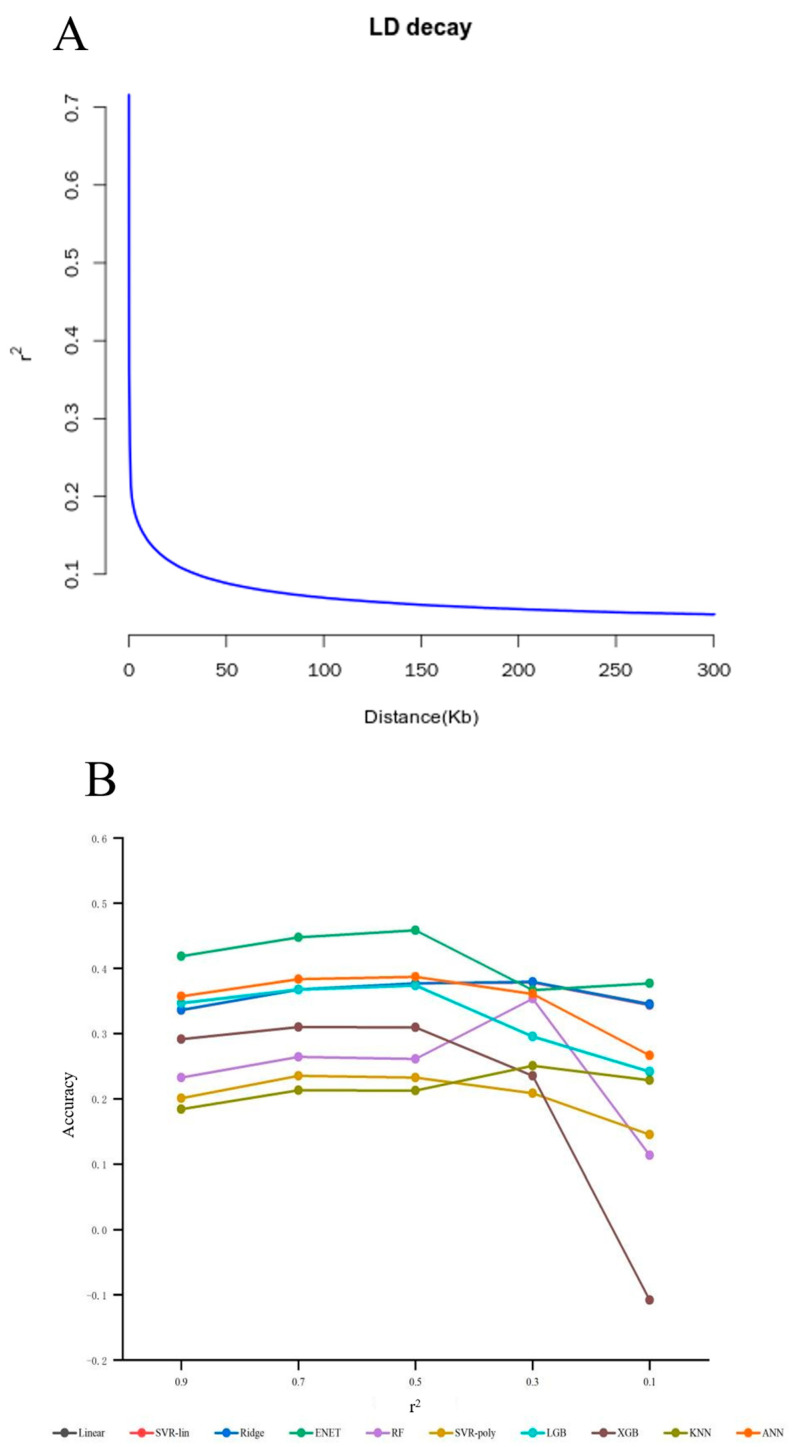
Predictive accuracy of gradient LD threshold screening SNP subset in candidate ML models. (**A**) LD decay diagram. (**B**) Predictive accuracy of the gradient LD threshold screening SNP subset in candidate ML models.

Genome-wide linkage disequilibrium (LD) decay analysis was conducted on the original genotype data of the reference population using PopLDdecay(v3.43) [37] software. The LD decay curve indicated that the squared correlation coefficient (r^2^) decreased to 0.1 at a genomic distance of approximately 25 kb (Figure 2A). Based on this result, a sliding window of 200 kb with a step size of 20 kb was applied for SNP filtering in subsequent analyses. Gradient LD pruning was then performed using PLINK 2.0 with varying r^2^ thresholds (0.9, 0.7, 0.5, 0.3, and 0.1). Prediction accuracies of various ML models exhibited an initial increase, followed by a decline as the r^2^ threshold decreased, achieving the best overall performance at r^2^ = 0.5 (Figure 2B and Figure S2 and Table S2). Nevertheless, even at this optimal threshold, the prediction accuracies remained significantly lower than those obtained through GWAS-based SNP selection, suggesting that relying solely on whole-genome LD pruning of resequencing data is suboptimal. This highlights the urgent need for more efficient SNP screening and feature selection strategies.

#### 3.1.3. GWAS + LD

In this study, we integrated LD analysis with GWAS to further optimize the SNP subset identified through GWAS screening, aiming to determine an optimal combined GWAS-LD filtering strategy for selecting SNPs associated with abdominal fat traits and thereby improve prediction accuracy.

As shown in Table 2, applying a moderately stringent GWAS *p*-value threshold (*p* < 1 × 10^−4^) significantly enhanced model prediction accuracy compared to using GWAS alone, whereas an overly strict threshold (*p* < 1 × 10^−5^) led to decreased performance. Following dual filtering incorporating LD pruning (with varying r^2^ thresholds), linear models (Linear, SVR-linear, Ridge, Elastic Net) and certain nonlinear models (SVR-polynomial, ANN) demonstrated superior GEBV prediction accuracy, making them suitable candidates for ensemble stacking. In contrast, RF, LightGBM, XGBoost, and KNN exhibited relatively lower accuracy. At a fixed LD threshold of r^2^ < 0.5, the linear models attained peak accuracy (0.9169) at a GWAS *p*-value threshold of 1 × 10^−4^, followed by a decline at stricter thresholds. Nonlinear models showed a similar pattern, with less pronounced improvement. Across various combinations, the highest average accuracy (0.8584) was achieved at *p* < 1 × 10^−4^combined with r^2^ < 0.1, resulting in a filtered set of 3549 SNPs selected as the optimal feature subset for downstream modeling. These findings indicate that joint GWAS and LD-based SNP screening outperforms GWAS alone by identifying SNP subsets with greater predictive power, providing a robust foundation for subsequent feature refinement and accurate genomic prediction modeling, and offering potential applicability for validation in independent populations.

### 3.2. Fine SNP Selection Based on Multiple Machine Learning Feature Selection Algorithms

To identify the optimal subset of SNP features associated with chicken abdominal fat traits, this study developed a two-stage feature selection framework that integrates both optimization and refinement processes. The framework combines the L1 regularization property of the Lasso model with a greedy optimization algorithm—RFE—to further refine SNP features, thereby improving prediction accuracy while managing computational costs. Starting from the SNP subset obtained via initial GWAS and LD analyses, we comparatively evaluated several feature selection methods to determine the most suitable algorithms for predicting the target traits. These included the Lasso model (L1 regularization, embedded method), Elastic Net (ENET, combining L1 and L2 regularization), Gradient Boosted Decision Trees (GBDT, a boosting-based model), RF, (based on bagging), the efficient RFE algorithm (a wrapper method), and an unsupervised autoencoder (AE) for dimensionality reduction.

As shown in Table 3, the Lasso model effectively compressed the high-dimensional SNP data, extracting 355 key SNPs with substantial influence on the traits. This selection improved the average prediction accuracy from 0.8666 (initial screening) to a maximum of 0.9198, achieving accuracies ranging from 0.7880 to 0.9818 across candidate models. Further refinement using RFE on the Lasso-selected 355 SNPs reduced the feature set to 177 SNPs (Table 4). The prediction accuracy of linear and some nonlinear models on this refined subset increased to a range of 0.8551 to 0.9956, with the average accuracy improving from 0.9198 to 0.9303. These results demonstrate that RFE successfully identified a core SNP subset with even greater predictive relevance and that the combined two-stage selection strategy outperformed either Lasso or RFE alone, providing a more optimal feature subset for the traits studied.

In summary, the Lasso + RFE feature selection framework leverages the complementary strengths of both methods to efficiently reduce the high-dimensional feature space into a concise, informative SNP subset. This high-quality input facilitates subsequent ML models in achieving superior genomic prediction performance.

### 3.3. Gene Annotation Results

**Figure 3:**
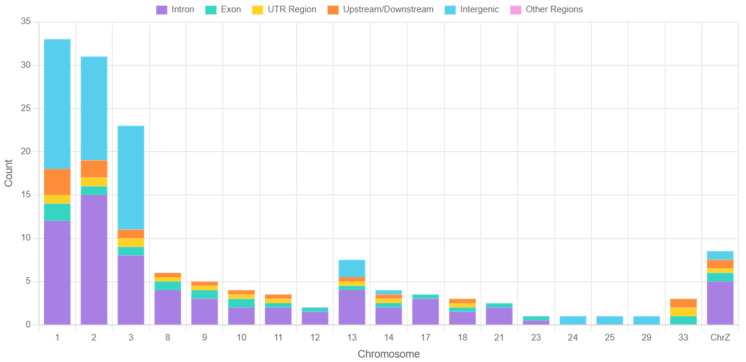
Annotation results. Kyoto Encyclopedia of Genes and Genomes (KEGG) and Gene Ontology (GO) pathway enrichment analyses were conducted on the identified genes.

A total of 177 key SNPs associated with chicken abdominal fat traits were identified in the reference population and subsequently annotated by comparison against the NCBI and Ensembl databases (Figure 3). Among these SNPs, 89 were located in intronic regions, 9 in exonic regions, 8 in 3′ untranslated regions (3′ UTR), 5 in 5′ untranslated regions (5′ UTR), 11 in upstream or downstream regions, and 55 in intergenic regions. Ultimately, these SNPs were annotated to 151 genes across the whole genome, of which 115 genes have been officially named (Table S9).

**Figure 4:**
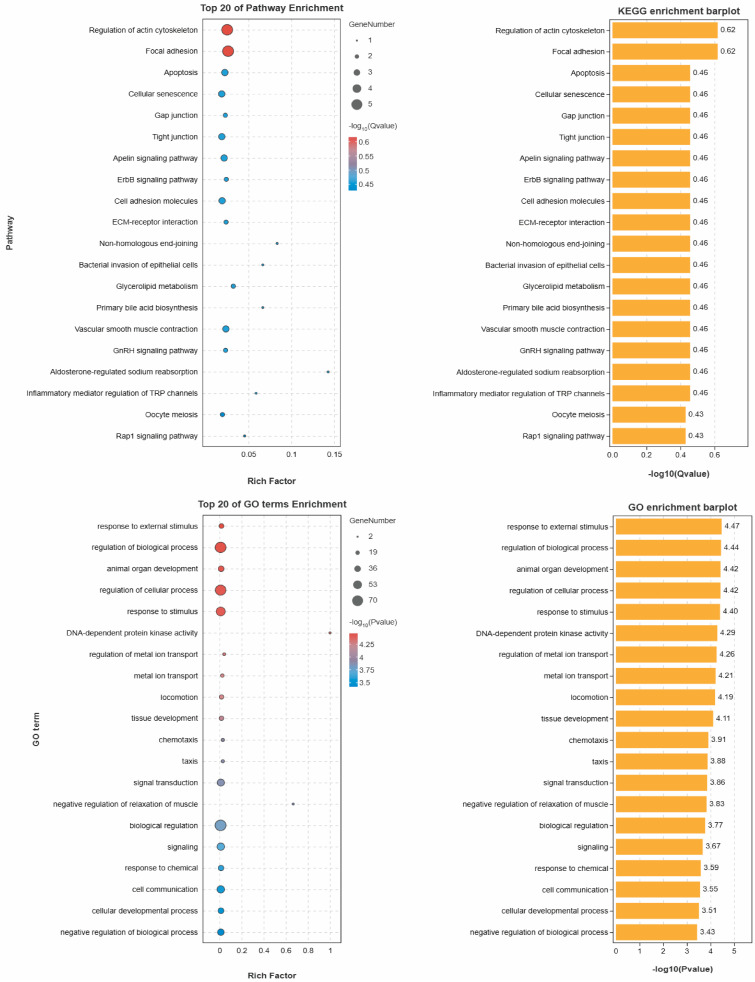
KEGG and GO analysis.

The KEGG enrichment results (Figure 4) indicate that pathways associated with adipose traits primarily regulate fat synthesis and accumulation through multiple mechanisms, including lipolysis, glucose metabolism, insulin sensitivity, inflammatory factor expression, and electrolyte homeostasis. Notably, the aldosterone-regulated sodium reabsorption pathway promotes adipogenesis by modulating sodium transport and globulinogenesis. Additionally, the Apelin signaling pathway influences fat deposition via effects on lipolysis and insulin sensitivity.

GO enrichment analysis revealed that these genes are significantly involved in biological processes such as negative regulation of biological activity, signal transduction, and cell development. These pathways may influence adipose traits by inhibiting the expression of adipogenic factors and promoting lipolytic activity, thus affecting fat synthesis and accumulation. Through meticulous screening and extensive analysis of the relevant literature, 41 genes closely related to fat metabolism and adipose traits were identified from the 151 candidate genes.

### 3.4. Comparison of Prediction Performance Between DAWSELF, Base Models, and Conventional Stacking Frameworks

**Figure 5:**
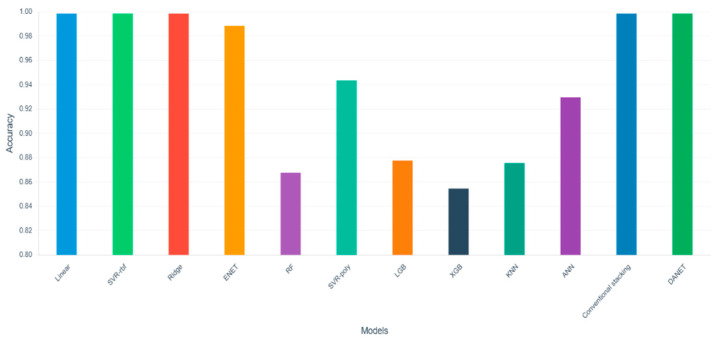
Comparison of genome prediction results of DAWSELF with base model and stacking frameworks.

Figure 5 and Table 5 demonstrate that the conventional stacking framework achieves a prediction accuracy of 0.9958 by integrating heterogeneous base models, supported by the strong accuracies of the individual base models (Linear: 0.9936; ENET: 0.9830; SVR-poly: 0.9404; ANN: 0.9296) and the meta-model (Ridge: 0.9937). This indicates that model integration facilitates knowledge fusion and enhances predictive performance. Notably, the DAWSELF framework further improves accuracy to 0.9965, surpassing the conventional stacked framework and confirming the superiority of its innovative design.

The DAWSELF framework achieves its highest prediction accuracy of 0.9965 using the refined set of 177 core SNP features, outperforming all individual base models as well as the conventional stacking integration. Overall, the optimized DAWSELF framework exhibits superior performance relative to both single base models and traditional integration methods. This model is a promising candidate for estimating breeding values of economically important traits, such as abdominal fat in broiler chickens, and holds potential for genomic prediction of other complex traits. Furthermore, this approach offers a practical analytical solution for ML-based genomic prediction using resequencing data.

### 3.5. Model Evaluation in Validation Populations

The genomic breeding value estimation framework developed in this study was validated using three independent validation populations, with predictive performance assessed across 10 ML models. The same SNP screening procedures—initial screening (GWAS + LD) followed by refined screening (Lasso + RFE)—were applied to the validation populations, as in the reference population (Tables S3–S8). The conventional stacking framework and the DAWSELF framework’s first-layer base models (Linear, ENET, SVR-poly, and ANN), along with Ridge as the meta-model in the second layer, were utilized for genomic estimated breeding value (GEBV) prediction.

Table 6 summarizes the prediction accuracies for abdominal fat weight traits in the three validation populations. In the G23 population, 780 core SNPs were retained after Lasso + RFE screening. The DAWSELF model achieved an accuracy of 0.9797, representing improvements of 0.0040, 0.0319, 0.0847, and 0.0072 over the constituent base models Linear, ENET, SVR-poly, and ANN, respectively. For the G27 population, 799 core SNPs were selected, and DAWSELF attained an accuracy of 0.9842, improving the Linear, ENET, SVR-poly, and ANN base models by 0.0026, 0.0208, 0.0628, and 0.0091, respectively. In the AA population, 878 core SNPs were identified. DAWSELF’s accuracy was 0.9882, with modest improvements of 0.0001, 0.0153, 0.0004, and 0.0009 over the respective base models. Although the magnitude of improvement in the AA population was smaller compared to the other two populations, DAWSELF consistently outperformed the conventional stacking model across all three populations. This demonstrates the effectiveness of the dynamic weighted integration strategy. Despite some variation in model performance among populations, DAWSELF achieved the highest or second-highest accuracy in all cases, indicating strong robustness and generalizability.

## 4. Discussion

In this experiment, we first investigated the optimal SNP screening threshold for GWAS, varying the *p*-value cutoff from 0.2 to 1 × 10^−4^. As the number of SNPs decreased, the predictive performance of most models improved steadily, indicating that filtering out irrelevant and noisy SNPs enhanced the signal-to-noise ratio. This finding is consistent with previous studies that have demonstrated the importance of balancing the inclusion of informative SNPs while excluding noise to improve predictive accuracy [37]. However, when the threshold was set more stringently at *p* < 1 × 10^−5^, model performance declined, likely due to over-filtering that excluded important associated loci. This observation aligns with the work of Song et al., who showed that overly stringent thresholds can lead to the exclusion of valuable genetic information [38]. The optimal GWAS *p*-value threshold identified in this study lies between 1 × 10^−3^ and 1 × 10^−4^, corresponding to a moderate SNP subset size of approximately 8000 to 30,000. This range balances signal retention and noise reduction effectively, achieving superior genomic prediction accuracy. Our results are in agreement with Lopez-Cruz et al., who reported similar findings in their study on optimal SNP selection for genomic prediction [39]. Thresholds that are either too lenient or too stringent adversely affect prediction performance, providing a useful guideline for future GWAS and SNP selection.

Subsequently, SNPs were preliminarily screened using a combination of GWAS and LD analyses. Preliminary results showed that, for the raw SNP data obtained from whole-genome resequencing, GWAS outperformed feature selection algorithms such as Lasso, in identifying SNPs strongly associated with target traits. This finding is supported by previous studies, which have demonstrated that GWAS is particularly effective in identifying SNPs with large effects on complex traits [40]. Direct feature selection via Lasso or similar models was less effective, likely because GWAS explicitly tests the association significance of individual SNPs, favoring those with larger effects, whereas Lasso emphasizes linear trait–marker relationships. For complex traits influenced by many small-effect loci, GWAS’s sensitivity to individual effect sizes and correction for multiple comparisons makes it a more robust pre-screening method, whereas Lasso may struggle with the high dimensionality of SNP data. Thus, GWAS-based pre-screening facilitates building more stable and accurate GEBV models [41].

In this study, we adopted a sequential screening of GWAS followed by LD analysis, rather than the reverse. Comparative pre-experiments of GWAS + LD versus LD + GWAS demonstrated that LD + GWAS performed similarly to genome-wide LD filtering, but with poorer SNP selection quality; hence, we did not pursue this approach further. This observation is consistent with findings from Wray et al. [42], who showed that initial LD filtering can lead to the loss of important SNPs, especially in populations with extensive SNP linkage from multiple generations of directional selection. We hypothesize that, in populations with extensive SNP linkage from multiple generations of directional selection, initial LD filtering may discard important SNPs, compromising subsequent GWAS efficacy. In contrast, performing GWAS first prioritizes SNPs with larger individual effects, crucial for polygenic traits, and thus provides a stronger foundation for subsequent LD-based redundancy reduction. This approach is supported by Kang et al., who demonstrated that GWAS is particularly effective in identifying significant SNPs in complex genetic architectures, even in the presence of extensive linkage disequilibrium [43]. Overall, both pre-experimental evidence and theoretical considerations support the GWAS-then-LD approach as yielding a more accurate and reliable SNP subset conducive to constructing robust predictive models.

Additionally, other population genetic approaches such as Fst and principal component analyses were explored for SNP selection but did not match the effectiveness of the GWAS + LD strategy and were therefore not further evaluated.

Finally, we implemented a two-stage feature selection framework combining Lasso-based dimensionality reduction with RFE refinement. This approach leverages Lasso’s efficient sparse coding to reduce computational burden and employs RFE’s greedy heuristic search to identify key predictive features. While RFE alone is computationally expensive, applying it after Lasso pre-screening substantially reduces its complexity, making the combined strategy a practical and effective optimization scheme.

During feature selection, the regularization strength parameter *α* of the Lasso model, as well as the number of retained features in the RFE method, were adaptively optimized via grid search combined with cross-validation, rather than being fixed arbitrarily. Taking the Lasso model as an example, the theoretical range of *α* spans from 0 to +∞, but in practice, it typically lies between 0 and 1. A smaller *α* corresponds to weaker regularization and results in a larger number of selected features, whereas a larger *α* induces stronger regularization and fewer selected features. In this study, multiple *α* values were explored through grid search, and model performance on the validation set was assessed by cross-validation to automatically identify the optimal *α* that minimizes validation error. This optimized *α* ensures that the Lasso model selects the most informative subset of features for predicting the target traits. This approach is supported by the work of Vovk et al. [44], who demonstrated that adaptive optimization of α through grid search and cross-validation can significantly improve model performance. Similarly, for recursive feature elimination, the RFECV function was employed to autonomously determine the optimal number of features to retain via cross-validation, thereby mitigating the risk of suboptimal selection caused by manual parameter settings [45]. This data-driven hyperparameter tuning approach avoids subjective and potentially biased fixed parameter settings, allowing adaptive optimization tailored to different datasets and improving both predictive accuracy and generalization. This method is further validated by Varma and Simon, who showed that using cross-validation for hyperparameter tuning can lead to more robust and accurate models [46].

In the genomic prediction of chicken abdominal fat weight, the results indicated that linear ML models (Linear, SVR-lin, Ridge, ENET) significantly outperformed nonlinear models (RF, SVR-poly, LGB, etc.). This aligns with previous findings that linear models generally perform better when traits are controlled by numerous small-effect loci, while nonlinear models tend to excel in scenarios dominated by a few large-effect loci. Since abdominal fat weight is a typical polygenic trait controlled by many micro-effect loci, linear models more effectively capture the underlying SNP-phenotype relationships. When selecting models for stacking, a balance must be struck between individual model performance and diversity. Although linear and some nonlinear models performed well initially and were considered base learners, relying solely on a single model type with high accuracy may reduce diversity, limiting the stacking framework’s overall fitting and generalization capabilities. Exclusively stacking linear models risks underfitting due to their inherent similarity and inability to capture nonlinear relationships, thus neglecting complex interactions between features and the target variable. To enhance model diversity and improve both learning and generalization, at least one nonlinear model should be incorporated as a base learner to compensate for mismatches among models. Furthermore, although the SVR-lin model demonstrated the best individual performance, it shares high homogeneity with SVR-poly as both are variants of support vector machines. Such homogeneity can diminish the robustness and stability of the stacked framework. Therefore, linear regression was chosen over SVR-lin to maintain greater diversity among base models.

After extensive tuning and experimentation, the optimal combination of base models was determined to be Linear, ENET, SVR-poly, and ANN, with Ridge regression serving as the meta-model for integration. This combination was thoroughly evaluated among the various alternatives and yielded the highest trait prediction accuracy. Specifically, linear regression excelled in capturing strong linear relationships with high precision; Elastic Net effectively handled noise and data variability, enhancing robustness to imperfect data; and SVR-poly, with its polynomial kernel, captured complex nonlinear patterns. This is in line with the work of Chang and Lin, who demonstrated that SVR with polynomial kernels can effectively model nonlinear relationships [47]. ANN, as a foundational deep learning model, modeled intricate nonlinear relationships between features and traits. Ridge regression was selected as the meta-model due to its robustness and stability in aggregating diverse base learner outputs, thereby improving the overall accuracy and reliability of the trait predictions. This choice is justified by Hoerl and Kennard, who showed that ridge regression can effectively reduce variance and improve model stability [48].

Eventually, through careful design and rigorous experimentation, we developed an efficient and stable integration approach that employs linear models, elastic net regression, support vector machines, and artificial neural networks as base learners, with ridge regression serving as the meta-model for integration. This ensemble framework has achieved the best predictive performance to date. Validation in independent populations confirmed that systematic model screening and integration significantly outperformed any single model alone. This progressive strategy not only advances the current study but also offers valuable general guidance for constructing robust predictive models in genomic selection.

Abdominal fat weight is a key economic trait in chickens that has attracted considerable breeding interest. The high- and low-fat line populations, along with the AA commercial breeder population studied here, have undergone over 20 generations of intense selection, resulting in substantial genetic variation for abdominal fat weight. Heritability, a measure of the genetic contribution to phenotypic variance, was high in these populations, ranging from 0.55 to 0.74. This indicates that 55–74% of the phenotypic variation is attributable to genotypic differences and is therefore amenable to improvement through selection. Such high heritability provides a strong foundation for precise estimation of individual breeding values and enhances selection accuracy. This is consistent with the findings of Li et al., who demonstrated that high heritability traits are more responsive to selection [49]. Leveraging these characteristics, the optimized SNP marker subsets combined with the two-stage ML feature selection framework yielded genomic breeding value predictions with accuracies exceeding 0.97. This underscores the superiority of ML algorithms for high-dimensional SNP screening and optimization, which effectively capture complex genotype–phenotype relationships and enable highly accurate breeding value estimation [50].

It should be noted that the relatively high accuracies reported in this study (up to 0.995–0.997) represent apparent performance under the current validation framework and may therefore overestimate the true external predictive ability. The small sample size of the reference population (330 individuals) limited our ability to implement a fully nested cross-validation scheme, as further reduction of the training set would compromise model stability and generalization. Instead, we adopted an 80/20 split with five-fold cross-validation within the training set, which is a pragmatic compromise commonly used in genomic prediction studies with limited data. In addition, technical constraints—including a sequencing depth of 10× and less than 30% SNP overlap across populations—prevented us from freezing the pipeline on G19 and directly applying it to other populations. To mitigate these limitations, we applied the same feature selection and optimization procedure independently within each population and observed consistent results across G23, G27, and AA. While these accuracies should be interpreted with caution, the reproducibility across independent datasets, together with the biological relevance of the identified SNPs and pathways, supports the robustness and practical value of the proposed pipeline.

The DAWSELF framework proposed in this study consistently achieved genomic prediction accuracies above 0.97 across four populations, including high- and low-fat line broilers and AA broiler populations. This exceptional performance is supported not only by genetic factors but also by biological validation. Gene annotation of the SNPs used in the prediction models revealed enrichment of 41 genes functionally related to fat synthesis and metabolism, demonstrating that the models capture biologically meaningful signals. Unlike overfitting models that merely memorize training data without biological relevance, our results indicate meaningful explanatory power. Furthermore, the framework exhibited excellent generalization, achieving similarly high accuracies in independent validation populations, reinforcing its robustness, and minimizing concerns of overfitting. Cross-validation and penalized model training further mitigated overfitting risk. Additionally, to reduce redundancy, a linkage disequilibrium (LD)-based SNP filtering strategy retained only the most informative marker within each high-LD block, which likely accounts for differences in SNP marker combinations across the three homogeneous populations (G19, G23, and G27). Differences in SNP counts between populations also highlight the model’s adaptive capability in capturing population-specific genetic architectures.

In conclusion, the consistent validation of this method in terms of stability, biological significance, and generalization strongly supports the reliability and robustness of high-precision genomic predictions across diverse populations. These results highlight the model’s exceptional capacity for complex trait dissection and establish a solid foundation for its broader application and implementation in poultry breeding programs.

## 5. Conclusions

In this study, we developed an efficient genomic prediction framework for chicken abdominal fat traits by integrating joint GWAS and LD analysis, a two-stage SNP feature selection strategy (“Lasso dimensionality reduction + RFE refinement”), and a dynamic adaptive weighted stacking ensemble learning model (DAWSELF). This approach not only substantially improved the accuracy and robustness of genomic estimated breeding values (GEBV) but also provided a generalizable feature selection and ensemble learning strategy for genomic prediction of other complex traits.

## Figures and Tables

**Table 1:** Covariates and heritability for phenotypic correction in populations.

Population	Sample Size	Covariates	Heritability (*h*^2^)
G19	330	Line, Weight	0.74
G23	439	Line, Sex, Weight	0.69
G27	446	Line, Weight	0.72
AA	400	Line, Sex, Weight	0.55

**Table 2:** Predictive accuracy of GWAS + LD combination scheme for screening SNP subset in candidate machine learning models.

GWAS(*p*)	LD(r^2^)	SNP Count	Linear	SVR-Lin	Ridge	ENET	RF	SVR-Poly	LGB	XGB	KNN	ANN	Mean
0.2	-	1,885,605	0.8485	0.8457	0.8454	0.5558	0.5181	0.7598	0.4041	0.3924	0.5254	0.8530	0.6548
	0.9	1,648,819	0.8496	0.8498	0.8496	0.5495	0.5187	0.7553	0.3934	0.4062	0.5284	0.8563	0.6557
	0.7	1,209,506	0.8556	0.8558	0.8557	0.5447	0.5163	0.7285	0.3800	0.4097	0.5293	0.8644	0.6540
	0.5	560,572	0.8600	0.8601	0.8601	0.5395	0.5271	0.7177	0.4089	0.4015	0.5091	0.8693	0.6553
	0.3	220,282	0.8545	0.8538	0.8543	0.5536	0.4806	0.6874	0.4152	0.4111	0.5030	0.8507	0.6464
	0.1	64,779	0.8507	0.8489	0.8492	0.5640	0.4346	0.6710	0.4440	0.3761	0.4985	0.8386	0.6376
0.1	-	1,019,085	0.8680	0.8683	0.8680	0.6090	0.5788	0.8359	0.5006	0.4632	0.6399	0.8684	0.7100
	0.9	891,723	0.8698	0.8699	0.8698	0.6098	0.5672	0.8333	0.4321	0.4726	0.6386	0.8625	0.7026
	0.7	651,664	0.8722	0.8724	0.8722	0.6101	0.5596	0.8286	0.3918	0.4619	0.6464	0.8793	0.6995
	0.5	314,163	0.8772	0.8779	0.8778	0.6108	0.5504	0.8206	0.3833	0.4524	0.6372	0.8847	0.6972
	0.3	134,124	0.8648	0.8650	0.8649	0.6421	0.5841	0.7976	0.4672	0.4013	0.5947	0.8740	0.6956
	0.1	42,253	0.8591	0.8590	0.8591	0.6945	0.5448	0.7737	0.5574	0.3495	0.5509	0.8565	0.6905
0.05	-	57,0318	0.8788	0.8792	0.8788	0.6641	0.6435	0.8706	0.5867	0.4948	0.6668	0.8779	0.7441
	0.9	499,542	0.8791	0.8795	0.8791	0.6774	0.6396	0.8689	0.5788	0.5671	0.6677	0.8767	0.7514
	0.7	362,390	0.8808	0.8810	0.8808	0.6728	0.6217	0.8638	0.5701	0.5384	0.7380	0.8736	0.7521
	0.5	181,130	0.8847	0.8848	0.8847	0.6834	0.5982	0.8641	0.4294	0.5776	0.6880	0.8852	0.7380
	0.3	85,012	0.8821	0.8820	0.8821	0.7184	0.6147	0.8544	0.4386	0.5028	0.7154	0.8781	0.7369
	0.1	31,104	0.8664	0.8667	0.8668	0.7669	0.5549	0.8395	0.4982	0.4245	0.7190	0.8666	0.7270
0.01	-	162,799	0.8960	0.8960	0.8958	0.8040	0.7351	0.8852	0.6457	0.6664	0.7327	0.8973	0.8054
	0.9	143,569	0.8966	0.8968	0.8968	0.8096	0.7302	0.8911	0.6368	0.6685	0.7364	0.8926	0.8055
	0.7	103,125	0.8983	0.8984	0.8983	0.8088	0.7146	0.8942	0.6176	0.6798	0.7641	0.8900	0.8064
	0.5	57,943	0.9006	0.9007	0.9007	0.8143	0.6994	0.8986	0.6006	0.6706	0.7823	0.8971	0.8065
	0.3	35,967	0.9034	0.9038	0.9038	0.8199	0.7035	0.8951	0.5849	0.6637	0.7838	0.8939	0.8056
	0.1	22,614	0.8924	0.8923	0.8924	0.8279	0.7704	0.8925	0.5473	0.6524	0.7855	0.8899	0.8043
1 × 10^−3^	-	33,738	0.9048	0.9051	0.9048	0.8785	0.7326	0.8705	0.6943	0.6445	0.7542	0.8968	0.8186
	0.9	30,336	0.9073	0.9074	0.9073	0.8813	0.7375	0.8738	0.6816	0.6497	0.7679	0.8961	0.8210
	0.7	21,777	0.9104	0.9105	0.9104	0.8847	0.7412	0.8866	0.6537	0.6515	0.8010	0.8957	0.8246
	0.5	14,594	0.9157	0.9158	0.9157	0.8880	0.7445	0.9046	0.6252	0.6564	0.8205	0.8955	0.8282
	0.3	11,871	0.9178	0.9180	0.9179	0.8884	0.7564	0.9065	0.5949	0.6748	0.8213	0.9029	0.8299
	0.1	10,400	0.9189	0.9191	0.9189	0.8879	0.7607	0.9075	0.5706	0.6845	0.8216	0.9096	0.8299
1 × 10^−4^	-	8345	0.9027	0.9031	0.9027	0.9055	0.7425	0.8473	0.7319	0.7958	0.7492	0.8839	0.8365
	0.9	7613	0.9060	0.9065	0.9060	0.9070	0.7636	0.8600	0.7344	0.7641	0.7716	0.8870	0.8406
	0.7	5505	0.9092	0.9096	0.9092	0.9081	0.7904	0.8871	0.7285	0.7524	0.8388	0.8878	0.8521
	0.5	4151	0.9161	0.9169	0.9161	0.9110	0.8003	0.9062	0.7207	0.7452	0.8623	0.8888	0.8584
	0.3	3731	0.9188	0.9194	0.9188	0.9139	0.8008	0.9100	0.7310	0.7749	0.8349	0.8965	0.8619
	0.1	3549	0.9209	0.9216	0.9209	0.9169	0.8024	0.9162	0.7315	0.8030	0.8322	0.9007	0.8666
1 × 10^−5^	-	2252	0.8172	0.8198	0.8173	0.8796	0.7432	0.7969	0.7705	0.7079	0.7241	0.8085	0.7885
	0.9	2066	0.8170	0.8191	0.8171	0.8845	0.7490	0.8025	0.7768	0.7146	0.7418	0.8015	0.7924
	0.7	1534	0.8243	0.8263	0.8244	0.8889	0.7624	0.8269	0.7857	0.7297	0.7618	0.7819	0.8012
	0.5	1268	0.8308	0.8332	0.8310	0.8882	0.7733	0.8574	0.7888	0.7350	0.8096	0.7664	0.8114
	0.3	1197	0.8206	0.8231	0.8208	0.8878	0.7722	0.8588	0.7926	0.7410	0.8110	0.8529	0.8181
	0.1	1168	0.8163	0.8195	0.8168	0.8875	0.7718	0.8653	0.8031	0.7430	0.7769	0.9195	0.8220

Note: “-“ indicates prediction results based solely on GWAS analysis in Figure 1. GWAS refers to the GWAS *p*-value threshold, with selection criterion *p* < the specified threshold. LD denotes the linkage disequilibrium coefficient threshold r^2^, with selection criterion r^2^ < the specified threshold. SNP Number is the count of SNPs retained after filtering. Linear stands for linear regression; SVR-lin, support vector regression with linear kernel; Ridge, ridge regression; ENET, Elastic Net; RF, Random Forest; SVR-poly, support vector regression with polynomial kernel; LGB, light gradient boosting machine; XGB, extreme gradient boosting; KNN, K-nearest neighbor; ANN, artificial neural network; and Mean represents the average prediction accuracy across candidate models.

**Table 3:** Comparison of model prediction performance under different feature selection methods.

Feature Selection Method	SNP Count	Linear	SVR-Lin	Ridge	ENET	RF	SVR-Poly	LGB	XGB	KNN	ANN	Mean
*p* < 1 × 10^−4^ + r^2^ < 0.1	3549	0.9209	0.9216	0.9209	0.9169	0.8024	0.9162	0.7315	0.8030	0.8322	0.9007	0.8666
Lasso	355	0.9818	0.9825	0.9821	0.9714	0.8471	0.9418	0.8540	0.7880	0.8882	0.9613	0.9198
ENET	1577	0.9696	0.9699	0.9696	0.9310	0.8210	0.9364	0.7851	0.8231	0.8798	0.9497	0.9035
GBDT	445	0.8699	0.8763	0.8704	0.9249	0.8493	0.9177	0.8218	0.8473	0.8924	0.8293	0.8699
RF	2833	0.9256	0.9261	0.9256	0.9225	0.8014	0.9196	0.7491	0.8123	0.8684	0.8987	0.8749
RFE	1769	0.9660	0.9664	0.9660	0.9280	0.8189	0.9326	0.7558	0.8087	0.8802	0.9366	0.8959
AE	10	0.9222	0.9199	0.9222	0.9237	0.8981	0.8832	0.8529	0.8510	0.8757	0.7661	0.8815

Note: *p* < 1 × 10^−4^ + r^2^ < 0.1 represents the initial SNP screening results combining GWAS and LD. Lasso refers to the linear L1-regularized Lasso model; ENET denotes the linear Elastic Net model with L1 and L2 regularization; GBDT is the nonlinear Gradient Boosting Decision Tree based on the boosting principle; RF stands for the nonlinear Random Forest model based on the bagging principle; RFE indicates recursive feature elimination using SVR-lin as the estimator; and AE is the unsupervised autoencoder.

**Table 4:** Comparison of model prediction performance under the Lasso + RFE secondary feature selection framework.

Feature Selection Method	SNP Count	Linear	SVR-Lin	Ridge	ENET	RF	SVR-Poly	LGB	XGB	KNN	ANN	Mean
Lasso	355	0.9818	0.9825	0.9821	0.9714	0.8471	0.9418	0.8540	0.7880	0.8882	0.9613	0.9198
Lasso + RFE	177	0.9936	0.9956	0.9937	0.9830	0.8655	0.9404	0.8746	0.8551	0.8721	0.9296	0.9303

**Table 5:** Comparison of genomic prediction accuracy between DAWSELF and stacking frameworks.

Models	Base Models	Meta Model	Accuracy
Conventional stacking	Linear, ENET, SVR-poly, ANN	Ridge	0.9958
DAWSELF	The first layer is the same as conventional stacking, with each subsequent layer independently selecting base models.	Ridge	0.9965

**Table 6:** Comparison of genomic prediction accuracy between DAWSELF and stacking frameworks in the validation population.

Validation Population	SNP Selection Stage	SNP Count	Linear	SVR-Lin	Ridge	ENET	RF	SVR-Poly	LGB	XGB	KNN	ANN	Means	Conventional Stacking	DAWSELF
G23	GWAS + LD	8998	0.9107	0.9111	0.9107	0.8767	0.6458	0.8204	0.5784	0.5569	0.4755	0.9076	0.7594		
	Lasso	864	0.9696	0.9695	0.9696	0.9409	0.7029	0.8848	0.6416	0.6215	0.5547	0.9541	0.8209		
	Lasso + RFE	780	0.9757	0.9756	0.9757	0.9478	0.7290	0.8950	0.6629	0.5057	0.5464	0.9725	0.8186	0.9770	0.9797
G27	GWAS + LD	6298	0.9124	0.9127	0.9124	0.8541	0.6454	0.8239	0.5717	0.5954	0.6000	0.9227	0.7751		
	Lasso	965	0.9703	0.9703	0.9703	0.9502	0.7005	0.9114	0.7079	0.6689	0.6255	0.9639	0.8439		
	Lasso + RFE	799	0.9816	0.9818	0.9816	0.9634	0.6926	0.9214	0.7385	0.6748	0.6973	0.9751	0.8608	0.9828	0.9842
AA	GWAS + LD	6649	0.9742	0.9743	0.9743	0.8973	0.8565	0.9517	0.7820	0.6416	0.8354	0.9693	0.8857		
	Lasso	891	0.9878	0.9880	0.9878	0.9723	0.8492	0.9875	0.7761	0.7223	0.8736	0.9857	0.9130		
	Lasso + RFE	878	0.9881	0.9882	0.9881	0.9729	0.8596	0.9878	0.7865	0.7246	0.8709	0.9873	0.9154	0.9871	0.9882

## Data Availability

All data is included in this paper.

## Supplementary Materials

The following supporting information can be downloaded at: https://www.mdpi.com/article/10.3390/ani15192843/s1; Figure S1: Average predictive accuracy of gradient GWAS threshold screening SNP subsets in candidate machine learning models; Figure S2: Average predictive accuracy of gradient LD threshold screening SNP subsets in candidate machine learning models; Table S1: Predictive accuracy of G19 population gradient GWAS threshold screening of SNP subset in machine learning models; Table S2: Predictive accuracy of G19 population gradient LD threshold screening of SNP subset in machine learning models; Table S3: Predictive accuracy of G23 population gradient GWAS threshold screening of SNP subset in machine learning models; Table S4: Predictive accuracy of G23 population GWAS + LD screening SNP subset in machine learning models; Table S5: Predictive accuracy of G27 population gradient GWAS threshold screening of SNP subset in machine learning models; Table S6: Predictive accuracy of G27 population GWAS + LD threshold screening of SNP subset in machine learning models; Table S7: Predictive accuracy of AA population gradient GWAS threshold screening of SNP subset in machine learning models; Table S8: Predictive accuracy of AA population GWAS + LD threshold screening of SNP subset in machine learning models; Table S9: Important SNPs associated with fat traits in chickens; Table S10: Literature mining-based association SNPs and genes for fat traits.

## References

[1] Smith K., Watson A.W., Lonnie M., Peeters W.M., Oonincx D., Tsoutsoura N., Simon-Miquel G., Szepe K., Cochetel N., Pearson A.G. (2024). Meeting the Global Protein Supply Requirements of a Growing and Ageing Population. Eur. J. Nutr..

[2] Saxena V.K., Kolluri G., Saxena V.K., Kolluri G. (2018). Selection Methods in Poultry Breeding: From Genetics to Genomics. Application of Genetics and Genomics in Poultry Science.

[3] Meuwissen T.H., Hayes B.J., Goddard M.E. (2001). Prediction of Total Genetic Value Using Genome-Wide Dense Marker Maps. Genetics.

[4] Daetwyler H.D., Pong-Wong R., Villanueva B., Woolliams J.A. (2010). The Impact of Genetic Architecture on Genome-Wide Evaluation Methods. Genetics.

[5] Habier D., Fernando R.L., Dekkers J.C.M. (2007). The Impact of Genetic Relationship Information on Genome-Assisted Breeding Values. Genetics.

[6] Endelman J.B. (2011). Ridge Regression and Other Kernels for Genomic Selection with R Package rrBLUP. Plant Genome.

[7] Habier D., Fernando R.L., Kizilkaya K., Garrick D.J. (2011). Extension of the Bayesian Alphabet for Genomic Selection. BMC Bioinf..

[8] Breiman L. (2001). Random Forests. Mach. Learn..

[9] Cortes C., Vapnik V. (1995). Support-Vector Networks. Mach. Learn..

[10] Krizhevsky A., Sutskever I., Hinton G.E. (2017). ImageNet Classification with Deep Convolutional Neural Networks. Commun. ACM.

[11] Xiang T., Li T., Li J., Li X., Wang J. (2023). Using Machine Learning to Realize Genetic Site Screening and Genomic Prediction of Productive Traits in Pigs. FASEB J. Off. Publ. Fed. Am. Soc. Exp. Biol..

[12] Mota L.F.M., Arikawa L.M., Santos S.W.B., Fernandes Júnior G.A., Alves A.A.C., Rosa G.J.M., Mercadante M.E.Z., Cyrillo J.N.S.G., Carvalheiro R., Albuquerque L.G. (2024). Benchmarking Machine Learning and Parametric Methods for Genomic Prediction of Feed Efficiency-Related Traits in Nellore Cattle. Sci. Rep..

[13] Faridi A., Sakomura N.K., Golian A., Marcato S.M. (2012). Predicting Body and Carcass Characteristics of 2 Broiler Chicken Strains Using Support Vector Regression and Neural Network Models. Poult. Sci..

[14] Liang M., Chang T., An B., Duan X., Du L., Wang X., Miao J., Xu L., Gao X., Zhang L. (2021). A Stacking Ensemble Learning Framework for Genomic Prediction. Front. Genet..

[15] Guo L., Sun B., Shang Z., Leng L., Wang Y., Wang N., Li H. (2011). Comparison of Adipose Tissue Cellularity in Chicken Lines Divergently Selected for Fatness. Poult. Sci..

[16] Gilmour A.R. (2015). ASReml User Guide Release 4.1 Structural Specification.

[17] Swami A., Jain R. (2013). Scikit-Learn: Machine Learning in Python. J. Mach. Learn. Res..

[18] Danecek P., Auton A., Abecasis G., Albers C.A., Banks E., DePristo M.A., Handsaker R.E., Lunter G., Marth G.T., Sherry S.T. (2011). The Variant Call Format and VCFtools. Bioinformatics.

[19] Chang C.C., Chow C.C., Tellier L.C., Vattikuti S., Purcell S.M., Lee J.J. (2015). Second-Generation PLINK: Rising to the Challenge of Larger and Richer Datasets. GigaScience.

[20] Browning B.L., Tian X., Zhou Y., Browning S.R. (2021). Fast Two-Stage Phasing of Large-Scale Sequence Data. Am. J. Hum. Genet..

[21] Muthukrishnan R., Rohini R. LASSO: A Feature Selection Technique in Predictive Modeling for Machine Learning. Proceedings of the 2016 IEEE International Conference on Advances in Computer Applications (ICACA).

[22] Tibshirani R. (1996). Regression Shrinkage and Selection via the Lasso. J. R. Stat. Soc. B.

[23] Luts J., Ojeda F., Van de Plas R., De Moor B., Van Huffel S., Suykens J.A.K. (2010). A Tutorial on Support Vector Machine-Based Methods for Classification Problems in Chemometrics. Anal. Chim. Acta.

[24] Zou H., Hastie T. (2005). Regularization and Variable Selection via the Elastic Net. J. R. Stat. Soc. B.

[25] Geva S., Sitte J. (1991). Adaptive Nearest Neighbor Pattern Classification. IEEE Trans. Neural Netw..

[26] Schmidhuber J. (2015). Deep Learning in Neural Networks: An Overview. Neural Netw..

[27] Biau G., Scornet E. (2016). A Random Forest Guided Tour. Test.

[28] Schapire R.E., Denison D.D., Hansen M.H., Holmes C.C., Mallick B., Yu B. (2003). The Boosting Approach to Machine Learning: An Overview. Nonlinear Estimation and Classification.

[29] Friedman J.H. (2001). Greedy Function Approximation: A Gradient Boosting Machine. Ann. Stat..

[30] Chen T., Guestrin C. XGBoost: A Scalable Tree Boosting System. Proceedings of the 22nd ACM SIGKDD International Conference on Knowledge Discovery and Data Mining.

[31] Weinberger K.Q., Blitzer J., Saul L.K. (2005). Distance Metric Learning for Large Margin Nearest Neighbor Classification. Proceedings of the 19th International Conference on Neural Information Processing Systems.

[32] Isabelle G., André E. (2003). An Introduction to Variable and Feature Selection. J. Mach. Learn. Res..

[33] Lal T.N., Chapelle O., Weston J., Elisseeff A., Guyon I., Nikravesh M., Gunn S., Zadeh L.A. (2006). Embedded Methods. Feature Extraction: Foundations and Applications.

[34] Kohavi R., John G.H. (1997). Wrappers for Feature Subset Selection. Artif. Intell..

[35] Hinton G.E., Salakhutdinov R.R. (2006). Reducing the Dimensionality of Data with Neural Networks. Science.

[36] Zhou X., Stephens M. (2012). Genome-Wide Efficient Mixed Model Analysis for Association Studies. Nat. Genet..

[37] Zhang C., Dong S.-S., Xu J.-Y., He W.-M., Yang T.-L. (2019). PopLDdecay: A Fast and Effective Tool for Linkage Disequilibrium Decay Analysis Based on Variant Call Format Files. Bioinformatics.

[38] Song H., Wang W., Dong T., Yan X., Geng C., Bai S., Hu H. (2025). Prioritized SNP Selection from Whole-Genome Sequencing Improves Genomic Prediction Accuracy in Sturgeons Using Linear and Machine Learning Models. Int. J. Mol. Sci..

[39] Lopez-Cruz M., de Los Campos G. (2021). Optimal Breeding-Value Prediction Using a Sparse Selection Index. Genetics.

[40] Stranger B.E., Stahl E.A., Raj T. (2011). Progress and Promise of Genome-Wide Association Studies for Human Complex Trait Genetics. Genetics.

[41] Zhang Y., Zhang M., Ye J., Xu Q., Feng Y., Xu S., Hu D., Wei X., Hu P., Yang Y. (2023). Integrating Genome-Wide Association Study into Genomic Selection for the Prediction of Agronomic Traits in Rice (*Oryza sativa* L.). Mol. Breed..

[42] Domingues V. (2013). Replication–Transcription Conflict Promotes Gene Evolution. Nat. Rev. Genet..

[43] Kang H.M., Zaitlen N.A., Wade C.M., Kirby A., Heckerman D., Daly M.J., Eskin E. (2008). Efficient Control of Population Structure in Model Organism Association Mapping. Genetics.

[44] Vovk V., Shafer G. (2005). Good Randomized Sequential Probability Forecasting Is Always Possible. J. R. Stat. Soc. Ser. B (Stat. Methodol.).

[45] Gomede E. Recursive Feature Elimination: A Powerful Technique for Feature Selection in Machine Learning. Medium, 2 September 2023. https://medium.com/the-modern-scientist/recursive-feature-elimination-a-powerful-technique-for-feature-selection-in-machine-learning-89b3c2f3c26a.

[46] Varma S., Simon R. (2006). Bias in Error Estimation When Using Cross-Validation for Model Selection. BMC Bioinf..

[47] Chang C.-C., Lin C.-J. (2011). LIBSVM: A Library for Support Vector Machines. ACM Trans. Intell. Syst. Technol..

[48] Hoerl A.E., Kennard R.W. (1970). Ridge Regression: Biased Estimation for Nonorthogonal Problems. Technometrics.

[49] Li B., Zhang N., Wang Y.-G., George A.W., Reverter A., Li Y. (2018). Genomic Prediction of Breeding Values Using a Subset of SNPs Identified by Three Machine Learning Methods. Front. Genet..

[50] Ahn E., Oh S., Botkin J., Bhatt J., Lee D., Magill C. (2025). Machine Learning Reveals Signatures of Transposable Element Activity Driving Agronomic and Phenolic Traits in Mutagenized Sorghum. Discov. Plants.

