# Supplementary material for: Optimization of Genomic Breeding Value Estimation Model for Abdominal Fat Traits Based on Machine Learning
Source: Animals (Basel). 2025 Sep 29;15(19):2843. doi: 10.3390/ani15192843 (PMC12523389; doi:10.3390/ani15192843)
Supplement: Supplementary file 1 [file animals-15-02843-s001.zip › animals-3844901-supplementary.pdf]

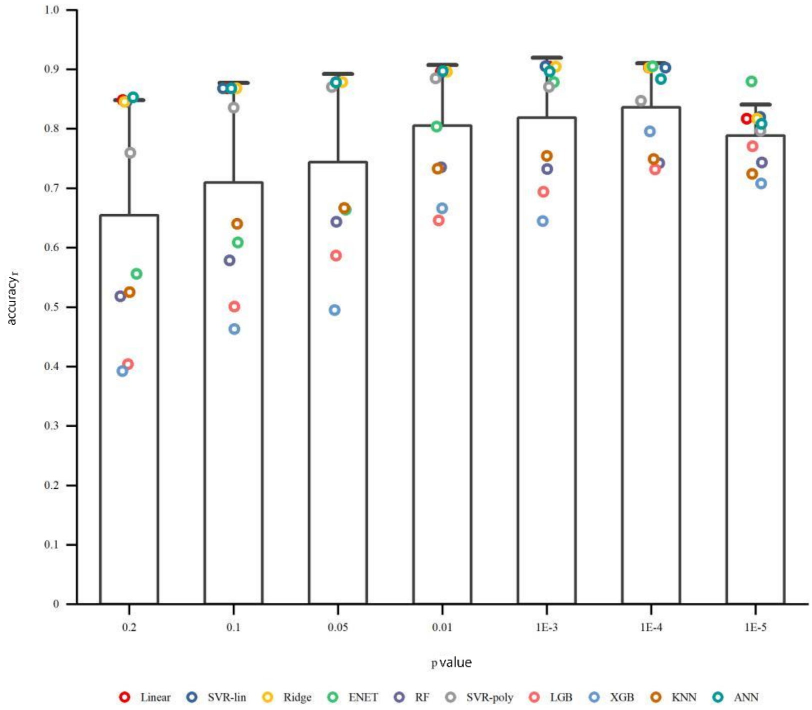

Figure S1: Average predictive accuracy of gradient GWAS threshold screening SNP subsets in candidate machine learning models

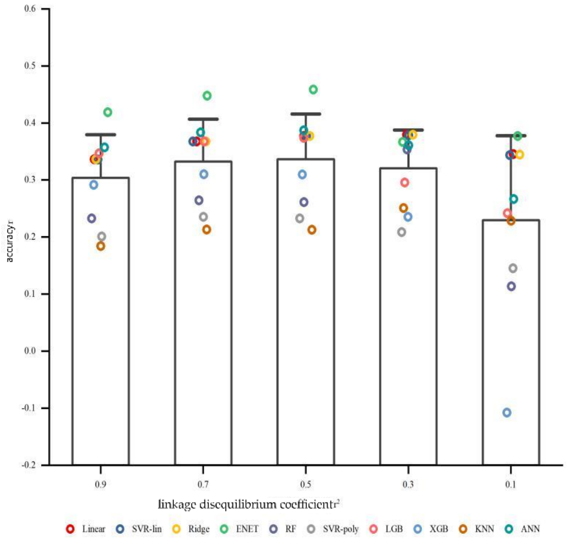

Figure S2: Average predictive accuracy of gradient LD threshold screening SNP subsets in candidate machine learning models

Table S1: Predictive accuracy of G19 population gradient GWAS threshold screening of SNP subset in machine learning models

GWAS(p)	SNP count	Linear	SVR-lin	Ridge	ENET	RF	SVR-poly	LGB	XGB	KNN	ANN	Mean
p < 0.2	1885605	0.8485	0.8457	0.8454	0.5558	0.5181	0.7598	0.4041	0.3924	0.5254	0.8530	0.6548
p < 0.1	1019085	0.8680	0.8683	0.8680	0.6090	0.5788	0.8359	0.5006	0.4632	0.6399	0.8684	0.7100
p < 0.05	570318	0.8788	0.8792	0.8788	0.6641	0.6435	0.8706	0.5867	0.4948	0.6668	0.8779	0.7441
p < 0.01	162799	0.8960	0.8960	0.8958	0.8040	0.7351	0.8852	0.6457	0.6664	0.7327	0.8973	0.8054
p < 1e-3	33738	0.9048	0.9051	0.9048	0.8785	0.7326	0.8705	0.6943	0.6445	0.7542	0.8968	0.8186
p < 1e-4	8345	0.9027	0.9031	0.9027	0.9055	0.7425	0.8473	0.7319	0.7958	0.7492	0.8839	0.8365
p < 1e-5	2252	0.8172	0.8198	0.8173	0.8796	0.7432	0.7969	0.7705	0.7079	0.7241	0.8085	0.7885

Table S2: Predictive accuracy of G19 population gradient LD threshold screening of SNP subset in machine learning models

LD（r2）	SNP count	Linear	SVR-lin	Ridge	ENET	RF	SVR-poly	LGB	XGB	KNN	ANN	Mean
r2 < 0.9	8454422	0.3361	0.3358	0.3361	0.4187	0.2324	0.2008	0.3467	0.2914	0.1842	0.3569	0.3039
r2 < 0.7	5719212	0.3674	0.3672	0.3674	0.4476	0.2640	0.2352	0.3674	0.3100	0.2131	0.3832	0.3322
r2 < 0.5	2152072	0.3768	0.3767	0.3770	0.4583	0.2611	0.2323	0.3737	0.3093	0.2126	0.3868	0.3365
r2 < 0.3	725642	0.3791	0.3782	0.3790	0.3666	0.3535	0.2084	0.2954	0.2352	0.2507	0.3608	0.3207
r2 < 0.1	200031	0.3452	0.3432	0.3443	0.3770	0.1133	0.1450	0.2417	-0.108	0.2283	0.2664	0.2296

Table S3: Predictive accuracy of G23 population gradient GWAS threshold screening of SNP subset in machine learning models

GWAS(p)	SNP count	Linear	SVR-lin	Ridge	ENET	RF	SVR-poly	LGB	XGB	KNN	ANN	Means
p < 0.05	557396	0.8649	0.8648	0.8649	0.2924	0.4997	0.7567	0.3410	0.2408	0.3391	0.8644	0.5929
p < 0.01	196311	0.8749	0.8747	0.8749	0.5381	0.5218	0.7753	0.5890	0.3744	0.3721	0.8654	0.6661
p < 1e-3	53160	0.8729	0.8728	0.8729	0.8463	0.6153	0.7880	0.6203	0.5118	0.4430	0.8622	0.7306
p < 1e-4	15683	0.8177	0.8199	0.8177	0.8551	0.6113	0.7809	0.6047	0.4851	0.5386	0.8066	0.7138
p < 1e-5	4681	0.6527	0.7156	0.6536	0.8236	0.6606	0.7631	0.6395	0.5629	0.5451	0.7922	0.6809

Table S4: Predictive accuracy of G23 population GWAS+LD screening SNP subset in machine learning models

GWAS+LD	SNP count	Linear	SVR-lin	Ridge	ENET	RF	SVR-poly	LGB	XGB	KNN	ANN	Means
1e-3	53160	0.8729	0.8728	0.8729	0.8463	0.6153	0.7880	0.6203	0.5118	0.4430	0.8622	0.7306
1e-3+0.9	34098	0.8826	0.8824	0.8826	0.8538	0.6328	0.7908	0.5814	0.4878	0.4229	0.8832	0.7300
1e-3+0.7	11859	0.9064	0.9068	0.9064	0.8684	0.6377	0.8230	0.5862	0.5435	0.4707	0.9127	0.7562
1e-3+0.5	9853	0.9108	0.9113	0.9108	0.8734	0.5960	0.8213	0.5797	0.5333	0.4885	0.9226	0.7548
1e-3+0.3	8998	0.9107	0.9111	0.9107	0.8767	0.6458	0.8204	0.5784	0.5569	0.4755	0.9076	0.7594
1e-3+0.1	8519	0.9072	0.9076	0.9072	0.8775	0.6162	0.8178	0.5326	0.5198	0.4708	0.9105	0.7467

Table S5: Predictive accuracy of G27 population gradient GWAS threshold screening of SNP subset in machine learning models

GWAS	SNP count	Linear	SVR-lin	Ridge	ENET	RF	SVR-poly	LGB	XGB	KNN	ANN	Means
p < 0.05	374731	0.8986	0.8983	0.8987	0.3767	0.5056	0.7797	0.4313	0.4520	0.5269	0.8993	0.6667
p < 0.01	105601	0.9088	0.9084	0.9088	0.5635	0.6244	0.7989	0.5255	0.5461	0.5321	0.8977	0.7214
p < 1e-3	19353	0.8940	0.8940	0.8940	0.8277	0.6547	0.7912	0.5601	0.5583	0.5725	0.9000	0.7546
p < 1e-4	4034	0.8414	0.8424	0.8414	0.8320	0.6192	0.7685	0.5955	0.5131	0.5700	0.8622	0.7285
p < 1e-5	956	0.6318	0.7285	0.6343	0.7592	0.5637	0.7191	0.5240	0.4610	0.4494	0.7623	0.6233

Table S6: Predictive accuracy of G27 population GWAS+LD threshold screening of SNP subset in machine learning models

GWAS+LD	SNP count	Linear	SVR-lin	Ridge	ENET	RF	SVR-poly	LGB	XGB	KNN	ANN	Mean
1e-3	19353	0.8940	0.8940	0.8940	0.8277	0.6547	0.7912	0.5601	0.5583	0.5725	0.9000	0.7546
1e-3+0.9	17794	0.8955	0.8950	0.8951	0.8295	0.6334	0.7922	0.5474	0.5648	0.5720	0.9031	0.7528
1e-3+0.7	13224	0.9005	0.9005	0.9005	0.8367	0.6392	0.8075	0.5333	0.5596	0.5590	0.9019	0.7539
1e-3+0.5	7998	0.9125	0.9126	0.9125	0.8439	0.6979	0.8290	0.5352	0.5370	0.5982	0.9179	0.7697
1e-3+0.3	6835	0.9126	0.9128	0.9127	0.8490	0.6540	0.8261	0.5823	0.5216	0.5819	0.9133	0.7666
1e-3+0.1	6298	0.9124	0.9127	0.9124	0.8541	0.6454	0.8239	0.5717	0.5954	0.6000	0.9227	0.7751

Table S7: Predictive accuracy of AA population gradient GWAS threshold screening of SNP subset in machine learning models

GWAS	SNP count	Linear	SVR-lin	Ridge	ENET	RF	SVR-poly	LGB	XGB	KNN	ANN	Means
$p < 0.05$	489399	0.9699	0.9698	0.9699	0.2960	0.6652	0.9479	0.3341	0.2881	0.7176	0.9593	0.7118
$p < 0.01$	100480	0.9722	0.9719	0.9722	0.6170	0.7244	0.9379	0.5538	0.4422	0.7325	0.9689	0.7893
$p < 1e-3$	9799	0.9698	0.9697	0.9698	0.8973	0.8385	0.9262	0.7722	0.6445	0.7893	0.9670	0.8744
$p < 1e-4$	885	0.9118	0.9145	0.9119	0.9233	0.8493	0.8895	0.7785	0.7669	0.7620	0.9238	0.8632
$p < 1e-5$	61	0.7608	0.7812	0.7614	0.7871	0.7355	0.7698	0.7345	0.7090	0.6831	0.6567	0.7379

Table S8: Predictive accuracy of AA population GWAS+LD threshold screening of SNP subset in machine learning models

GWAS+LD	SNP count	Linear	SVR-lin	Ridge	ENET	RF	SVR-poly	LGB	XGB	KNN	ANN	Mean
1e-3	9799	0.9698	0.9697	0.9698	0.8973	0.8385	0.9262	0.7722	0.6445	0.7893	0.9670	0.8744
1e-3+0.9	9201	0.9702	0.9701	0.9702	0.8979	0.8542	0.9307	0.7682	0.6637	0.7779	0.9653	0.8768
1e-3+0.7	8276	0.9718	0.9717	0.9718	0.8978	0.8625	0.9383	0.7083	0.6286	0.8313	0.9712	0.8753
1e-3+0.5	6649	0.9742	0.9743	0.9743	0.8973	0.8565	0.9517	0.7820	0.6416	0.8354	0.9693	0.8857
1e-3+0.3	5728	0.9743	0.9748	0.9749	0.9000	0.8486	0.9562	0.6812	0.5798	0.8320	0.9711	0.8693
1e-3+0.1	5196	0.9740	0.9740	0.9740	0.9032	0.8680	0.9573	0.7124	0.6356	0.8461	0.9698	0.8814

Table.S9: Important SNPs associated with fat traits in chickens

CHR	rs	rsID	REF/ALT	Region Annotation (30kb)	GeneID
1	14050220	rs312533280	C/T	Intergenic	SRPK2
1	14565461	rs734014574	G/A	3′UTR	CCDC71L
1	16576357	rs14792135	C/G	Intergenic between TBC1D22A and LOC107051637	
1	16576359	rs735368975	T/G	Intergenic between TBC1D22A and LOC107051637	
1	16576361	rs740130634	A/T	Intergenic between TBC1D22A and LOC107051637	
1	20450879	NA	A/G	Intergenic between LOC124417103 and SELENOO	
1	25496912	rs313304047	T/C	Intergenic	TFEC
1	27004084	rs14802843	T/G	Intergenic between TMEM168 and LOC101747665	TMEM168
1	27396089	rs312686990	A/G	Intergenic	DOCK4
1	32022805	rs736685593	C/A	Intergenic between LOC112531642 and LOC101749839	
1	38522257	rs15240876	A/C	Intergenic	NAV3
1	62938764	rs317457024	G/A	Exonic	PTPRO
1	64700691	rs316801185	C/T	Intergenic between AEBP2 and LOC101747667	
1	67358568	rs313013024	G/A	Downstream	LOC418209
1	67502717	rs734155411	A/G	Intergenic between LOC418209 and LOC101750507	LOC101750507
1	67908655	rs738800604	G/A	Intergenic	ITPR2
1	80587469	rs316139753	T/C	Downstream	CHD1L
1	88722474	rs316554504	A/C	Intergenic	PHLDB2
1	93282949	rs739664037	T/C	Intergenic between VGLL3 and LOC101747669	LOC101747669
1	104254047	rs732331393	C/T	3′UTR	GRIK1
1	106145932	rs315502695	T/C	Intergenic	RUNX1
1	122532598	rs317927952	A/G	Intergenic	GLRA2
1	122759603	rs14882812	A/G	Intergenic	LOC101748844
1	124217294	rs317914822	G/C	Intergenic	ARHGAP6

（To be continued on next page）

（Continued from previous page）

CHR	rs	rsID	REF/ALT	Region Annotation (30kb)	GeneID
2	115743762	rs315791086	G/A	Intergenic between C2H8ORF34 and LOC101747717	
2	116807784	NA	C/A	3′UTR	LOC124417715
2	117068585	rs737497617	G/C	Intergenic between TRPA1 and KCNB2	TRPA1
2	117255055	rs317187943	T/C	Intergenic	KCNB2
2	129028078	rs16136069	T/C	Intergenic	DCAF13
2	141047280	rs317773913	A/G	Downstream	LOC101750227
2	141047285	rs732444563	C/T	Downstream	LOC101750227
2	147421501	rs314103285	A/G	Intergenic between RHPN1 and MAFA	
2	147929880	NA	C/A	Exonic	LOC107052803
3	9804651	rs3385432817	C/A	5′UTR	VPS54
3	9902786	rs3385376595	G/A	Upstream	LOC112532189
3	18579244	rs312760145	G/T	3′UTR	SLC30A10
3	20454521	rs313973327	T/C	Intergenic between KCTD3 and KCNK2	KCTD3
3	21936798	rs16236326	G/A	Intergenic	RD3
3	22746283	rs739277147	A/G	Intergenic between PRPH2 and TBCC	PRPH2, TBCC
3	32300454	rs741426455	G/A	Intergenic between CRIM1 and LOC107052891	
3	33830099	rs736821998	G/A	Intergenic	SMYD3
3	47963590	rs315969886	C/T	Intergenic between LRP11 and PPP1R14C	LRP11
3	49305387	rs735443526	T/C	Intergenic	MTRF1L
3	49943286	rs16274526	T/C	Intergenic between CNKSR3 and SCAF8	
3	50052087	rs318238295	T/C	Intergenic	SCAF8
3	52084985	rs1060396909	G/A	Intergenic	PHACTR2
3	64952006	rs312678009	A/G	Intergenic between LOC107053094 and LOC112532117	LOC112532117
3	69239465	rs312892246	A/G	Intergenic between LOC10175 and LOC112532171	
3	74876182	rs318132121	C/T	Intergenic between LOC107053107 and BACH2	
3	81445029	rs313826713	G/A	Intergenic	LOC107053125
3	82027767	rs16311609	G/A	Intergenic between LOC112532113 and MIR30A	LOC112532113, MIR30A

（To be continued on next page）

（Continued from previous page）

CHR	rs	rsID	REF/ALT	Region Annotation (30kb)	GeneID
3	89882054	rs317258560	G/A	Intergenic between CSMD1 and LOC112532174	CSMD1
3	90238723	rs741463985	G/A	Intergenic between LOC112532174 and MYOM2	
3	95785382	rs316220754	G/C	Intergenic	MBOAT2
3	97499766	rs1058572005	C/T	Intergenic	LOC112532124
3	99517571	rs316015419	T/G	Intergenic between FAM49 and LOC107051696	
4	11171019	rs316337047	T/C	Intergenic	SLAINL
4	16489860	rs1058246613	C/T	Upstream, Downstream	NKRF, SEPT6
4	24556932	rs316846222	G/C	3’UTR	DDX60
4	31872081	rs731048815	A/C	Intergenic	NR3C2
4	33367464	rs16387716	C/T	Intergenic	FBXW7
4	33691039	rs16388321	C/A	Intergenic	CSGALNACT1
4	37080684	rs736711521	T/G	Intergenic	COL25A1
4	38047740	rs317539256	T/A	Exonic	TBCK
4	38329446	rs15547552	T/C	Intergenic	LOC107051759
4	45729733	rs741123154	T/C	Intergenic	ARHGAP24
4	47799001	rs314019994	T/C	Intergenic	ADGRL3
4	60825905	rs733012800	C/T	Intergenic	LOC112532412
4	72466562	rs736593571	A/G	Intergenic between LOC112532445 and STIM2	LOC112532445
4	77192262	rs313357228	G/A	Intergenic between RAB28 and LOC112532323	LOC112532323
5	11830354	rs740618814	A/T	Intergenic	KCNC1
5	12012057	rs313367088	G/A	Intergenic	SERGEF
5	13383512	rs316518128	T/C	Exonic	LOC107053399
5	39313107	rs739354921	G/A	Intergenic	NRXN3
5	46714021	NA	C/A	Intergenic between LOC124417995 and LOC124417996	
6	4106644	rs733954210	T/C	Intergenic between LOC101751438 and NRG3	NRG3
6	4470302	rs734699249	A/T	Intergenic	NRG3

（To be continued on next page）

（Continued from previous page）

CHR	rs	rsID	REF/ALT	Region Annotation (30kb)	GeneID
6	5168326	rs317966931	A/C	Intergenic between SFTPA2 and MBL2	SFTPA2, MBL2
6	6427216	rs317791505	C/G	Intergenic between LOC101748176 and LOC107053623	LOC107053623
6	31359312	rs312648495	C/T	Downstream	PLPP4
6	33780830	rs731451502	A/G	Intergenic	DOCK1, FAM196A
7	9465208	rs313343258	C/T	Intergenic	HECW2
7	12093033	rs739164214	A/G	Intergenic	ADAM23
7	16541896	rs1059287245	G/A	Intergenic	CIR1
7	26892159	rs315625464	C/T	Intergenic	MYLK
8	6564705	rs736527281	G/A	Intergenic	RASAL2
8	15281300	rs739326902	A/T	Intergenic	LOC101750715
8	17591304	rs313609211	C/A	Intergenic between LOC107053990 and MIR1620	
8	18523297	rs315717075	T/G	Intergenic between LOC112532906 and GIPC2	GIPC2
8	18539512	rs13681165	G/T	Intergenic	GIPC2
8	25486511	rs314730673	C/T	Intergenic	C8H1orf168
8	28072829	rs317426819	G/A	Intergenic	PDE4B
9	4121400	rs732056523	T/C	5′UTR	AMOTL2
9	20389169	rs316123003	A/T	Intergenic between ZBBX and LOC112533015	ZBBX
9	21021665	rs313666419	A/G	Intergenic	LOC101749407
9	21556605	rs736247117	G/A	Exonic	LOC107054138
9	21990282	rs735107136	C/T	Intergenic	SCHIP1
9	22154063	rs318003896	T/C	Intergenic	RARRES1
10	2791036	rs14940221	G/A	Intergenic	LINGO1
10	2848617	rs737312881	G/A	Intergenic	LINGO1
10	18409966	rs312509371	A/G	Intergenic	MEGF11
10	18494672	rs316998160	C/T	Upstream	MAP2K1, TIPIN
11	1565739	rs732220432	C/T	Upstream	HYDIN, LOC112533290

（To be continued on next page）

（Continued from previous page）

CHR	rs	rsID	REF/ALT	Region Annotation (30kb)	GeneID
11	4806874	rs731658547	A/C	Intergenic between CHD9 and LOC112533305	LOC112533305
11	10051017	rs14743539	T/A	Intergenic	PEPD
11	14122983	rs15621180	C/T	Intergenic	WWOX
12	3046880	NA	C/G	Intergenic	LOC101747452
12	17032476	rs314062004	G/A	Intergenic	LOC107054413
13	3709789	rs731773786	C/G	Intergenic	DOCK2
13	3753444	rs738035865	A/G	Intergenic	DOCK2
13	3884368	rs316488940	C/T	Intergenic	SLIT3
13	3885068	rs315381897	T/A	Intergenic	SLIT3
13	11619279	rs3388164867	C/A	Exonic	GEMIN5
13	14987543	rs313342105	T/A	Intergenic between NEUROG1 and LOC107054564	NEUROG1, LOC107054564
13	15125398	rs80665670	G/C	Intergenic between LOC107054563 and PITX1	
14	193852	rs313266312	C/T	Intergenic	SNX29
14	4024714	rs14072263	G/A	Intergenic	RADIL
14	11604964	rs733162152	T/C	Intergenic	RBFOX1
14	14764369	rs315132655	T/C	Intergenic between GPR139 and GPRC5B	GPRC5B
17	16933	NA	C/A	Intergenic	LOC124417279
17	5918819	rs732887153	C/T	Intergenic	LOC112529987
17	6972829	rs737875410	G/A	Intergenic	MED27
17	11020479	rs314676452	C/T	Intergenic	GARNL3
18	1858893	rs313402514	T/G	Intergenic between DNAH9 and LOC112530020	DNAH9 LOC112530020
18	6392379	rs735619324	A/C	Intergenic between LOC107052251 and TOM1L1	
18	10929526	rs313948732	G/A	Intergenic	CACNA1G
18	11060099	rs15470517	G/A	Upstream	RAB37
21	4978302	rs738241362	C/T	Intergenic	KAZN

（To be continued on next page）

（Continued from previous page）

CHR	rs	rsID	REF/ALT	Region Annotation (30kb)	GeneID
21	5005204	rs737748705	A/G	Intergenic	KAZN
21	5025456	rs314588982	T/C	Intergenic	KAZN
23	4680772	rs739063057	A/G	Intergenic	KIAA0319L
24	4359211	rs1057966550	T/C	Exonic	COLCA2
25	1941692	rs3387272223	A/G	Exonic	IL6R
29	366073	NA	C/G	Intergenic between LOC121107770 and LOC121107782	
33	1565289	NA	G/T	Intergenic	LOC121107968
33	1587662	NA	A/C	Intergenic between LOC121107970 and LOC100858567	
33	2865343	NA	A/C	3′UTR	LOC776171
33	2865344	NA	G/T	3′UTR	LOC776171
ChrZ	2397197	rs734804256	C/T	Intergenic	RNF165
ChrZ	6442922	rs738967277	T/G	Intergenic between LOC101751865 and CELF4	
ChrZ	17205700	rs314396687	T/G	Intergenic	GZMA
ChrZ	31839350	rs737802418	G/A	Intergenic	NFIB
ChrZ	60403592	rs312369704	C/T	Intergenic	ADGRV1
ChrZ	61402245	NA	G/A	Intergenic between LOC100859691 and TMEM161B	
ChrZ	65790355	rs737129121	T/G	Upstream	RNF20
ChrZ	84313171	rs740615521	T/G	Intergenic between LOC112530646 and ZNF608	ZNF608

Table.S10: Literature mining-based association SNPs and genes for fat traits

CHR	rs	rsID	Region Annotation	GeneID
1	14050220	rs312533280	Intergenic	SRPK2
1	62938764	rs317457024	Exonic	PTPRO
1	67908655	rs738800604	Intergenic	ITPR2
1	104254047	rs732331393	3'UTR	GRIK1
1	106145932	rs315502695	Intergenic	RUNX1
1	180125551	rs313887054	Intergenic between KDELC2 and ATM	ATM
2	20055183	rs3385019602	5'UTR	PTER
2	20243293	rs738072003	5'UTR	ITGA8
2	27862927	rs317883849	Intergenic	AGMO
2	109656872	rs316377371	Intergenic	RB1CC1
2	112631630	rs737619772	Intergenic	CHD7
2	114098284	rs738316703	3'UTR	CYP7B1
2	114098284	rs738316703	3'UTR	TRPA1
3	18579244	rs312760145	3'UTR	SLC30A10
3	82027767	rs16311609	Intergenic between LOC112532113 and MIR30A	MIR30A
3	95785382	rs316220754	Intergenic	MBOAT2
4	31872081	rs731048815	Intergenic	NR3C2
4	33367464	rs16387716	Intergenic	FBXW7
4	38047740	rs317539256	Exonic	TBCK
5	39313107	rs739354921	Intergenic	NRXN3
6	4106644	rs733954210	Intergenic between LOC101751438 and NRG3	NRG3
6	4470302	rs734699249	Intergenic	
6	5168326	rs317966931	Intergenic between SFTPA2 and MBL2	MBL2
6	31359312	rs312648495	Downstream	PLPP4
7	12093033	rs739164214	Intergenic	ADAM23
7	26892159	rs315625464	Intergenic	MYLK

（To be continued on next page）

(Continued from previous page)

CHR	rs	rsID	Region Annotation	GeneID
8	6564705	rs736527281	Intergenic	RASAL2
8	18523297	rs315717075	Intergenic between LOC112532906 and GIPC2	GIPC2
8	18539512	rs13681165	Intergenic	
8	28072829	rs317426819	Intergenic	PDE4B
9	4121400	rs732056523	5′UTR	AMOTL2
9	22154063	rs318003896	Intergenic	RARRES1
10	2791036	rs14940221	Intergenic	LINGO1
10	2848617	rs737312881		
10	18494672	rs316998160	Upstream	MAP2K1
11	10051017	rs14743539	Intergenic	PEPD
11	14122983	rs15621180	Intergenic	WWOX
13	3709789	rs731773786	Intergenic	DOCK2
13	3753444	rs738035865		
13	3884368	rs316488940	Intergenic	SLIT3
13	3885068	rs315381897		
14	14764369	rs315132655	Intergenic between GPR139 and GPRC5B	GPRC5B
18	11060099	rs15470517	Upstream	RAB37
25	1941692	rs3387272223	Exonic	IL6R
ChrZ	65790355	rs737129121	Upstream	RNF20
ChrZ	84313171	rs740615521	Intergenic between LOC112530646 and ZNF608	ZNF608
